# Immune-Related Proteomic Profiling in Clear Cell Renal Cell Carcinoma: Tissue and Soluble Biofluid Biomarkers

**DOI:** 10.3390/ijms27219764

**Published:** 2026-10-30

**Authors:** Christina Anatoliotaki, Sevastiani Antonouli, Vassilios Tzortzis, Panagiotis J. Vlachostergios, Ioannis Zachos, Eleni Thodou, Antonis Giakountis, Maria Samara

**Affiliations:** 1 Department of Biochemistry and Biotechnology, School of Health Sciences, University of Thessaly, 41500 Larissa, Greece; 2 Department of Histology and Embryology, Faculty of Medicine, School of Health Sciences, University of Thessaly, 41500 Larissa, Greece; 3 Department of Urology, Faculty of Medicine, University Hospital of Larissa, University of Thessaly, 41500 Larissa, Greece; 4 Department of Medical Oncology, Faculty of Medicine, School of Health Sciences, University Hospital of Ioannina, University of Ioannina, 45110 Ioannina, Greece; 5 Division of Hematology & Medical Oncology, Weill Cornell Medicine, New York, NY 10065, USA; 6 Department of Pathology, Faculty of Medicine, School of Health Sciences, University of Thessaly, 41500 Larissa, Greece

**Keywords:** clear cell renal cell carcinoma, immune-related proteomic biomarkers, immune checkpoint proteins, liquid biopsy

## Abstract

Clear cell renal cell carcinoma (ccRCC) shows complex interactions with the tumor microenvironment, involving a range of immune checkpoint proteins and circulating biomarkers that may influence disease progression and response to treatment. Recent technological advances have identified tissue-based and circulating immune-related proteomic targets such as programmed death-ligand 1 (PD-L1), cytotoxic T-lymphocyte-associated protein 4 (CTLA-4), and circulating interleukin-8 (cIL-8) as potential diagnostic, prognostic and predictive biomarkers. Despite significant progress, ccRCC remains a major clinical challenge due to its diverse molecular and immunological characteristics that hinder the clinical utility of these biomarkers. The present review provides an updated overview of the current evidence regarding immune-related proteomic biomarkers in ccRCC, including both tissue-derived and circulating proteins. Herein, we discuss the potential diagnostic, prognostic, and predictive value of such agents, emphasizing their biological significance and potential to guide personalized immunotherapeutic strategies. Particular attention is given to the developing liquid-biopsy-based techniques and their application as novel minimally invasive methods of identifying new targets for disease monitoring and treatment evaluation. Finally, we focus on emerging therapeutic approaches and combined strategies, highlighting how biomarker-driven precision oncology continues to reshape ccRCC management.

## 1. Introduction

Renal cell carcinoma (RCC), also historically known as hypernephroma or Grawitz tumor, is the most prevalent type of kidney cancer, accounting for 90% of all primary renal malignancies in adults [1]. Approximately 440,000 new cases of RCC are diagnosed each year globally, marking this form of the disease as the 14th most common type of cancer [2]. By 2050, 745,791 new cases and 304,861 new deaths are expected [3]. Depending on the geographic area, the 5-year overall survival (OS) rate ranges from 40% to 75% [3]. In Europe, RCC affects more men than women and it is typically diagnosed between 65 and 70 years of age in men and over 70 years in women. 

Unlike kidney cancer that involves the renal pelvis or renal medulla, RCC develops from the epithelial lining of the kidney bed, such as in the renal tubules (ccRCC: clear cell renal cell carcinoma, arising from the epithelial cells of the proximal renal tubules; pRCC: papillary renal cell carcinoma arising from the epithelial cells of the distal renal tubules; and chRCC: chromophobe renal cell carcinoma arising from the intercalated cells of the collecting duct) [4]. About 20 years ago, RCC was categorized into clear and granular cell variants, while the World Health Organization (WHO) classified RCC in three morphologically distinct histological subtypes known as clear cell (ccRCC), papillary (pRCC), and chromophobe (chRCC). Histologically, ccRCC may originate in the Bowman capsule, as shown from mouse model studies [5,6]. In the 5th edition of 2022, RCC classification was redefined based on a molecularly driven renal tumor criteria [6]. However, no substantial changes were made to the classification of ccRCC, which still remains the most common subtype, accounting for approximately 75% of diagnoses followed by pRCCC at 15% and chromophobe chRCC at 5% [6,7]. While non-clear cell RCCs represent a heterogeneous group with unique molecular characteristics, ccRCC has been the primary focus of translational research due to its high incidence and remarkable immunogenicity.

Hereditary cases account for about 5% of all RCC cases and are caused by germline variants [8]. Pathogenic germline variants in a variety of gene loci such as *VHL*, *ELOC*, *TSC 1/2*, *MET*, *FLCN*, *PRDM10*, *SDH A/B/C/D*, *MiTF*, *CDC73*, *FH*, *PTEN*, *BAP1*, *SMARCB1*, *CHEK2*, *MUTYH*, *BRCA2*, *ATM*, and *APC* are associated with an increased predisposition to RCC [3,9]. In ccRCC, the vast majority (95%) are sporadic, while 5% are linked to hereditary syndromes such as von Hippel–Lindau disease or tuberous sclerosis [4]. However, both hereditary and sporadic *VHL* gene alterations/mutations are widely accepted as a hallmark of ccRCC [10,11]. In fact, approximately 95% of ccRCCs demonstrate cytogenetic alterations like a loss of the short arm of chromosome 3 (3p), where *VHL* resides (3p25.3). Others include gain of 5q (69%), partial loss of 14q (42%), 7q gain (20%), 8p deletion (32%), and 9p loss (29%) [12,13]. These are commonly associated with pathogenic variants in chromatin remodeling genes *PBRM1* (40%), *SETD2* (15%), and *BAP1* (15%) [14]. This molecular pathogenesis of ccRCC leads to elevated protein stability of hypoxia-inducible factor 1 alpha and hypoxia-inducible factor 2 alpha (HIF-1α and HIF-2α), dysregulated angiogenesis, metabolic reprogramming, and substantial remodeling of the tumor microenvironment (TME) [13,15]. 

The TME, composed of immune cells, vasculature, signaling molecules, and extracellular matrix plays a pivotal role in anti-tumor immunity, consequently affecting sensitivity to immunotherapy as well as therapeutic outcome [16,17]. The TME regulates tumor immune surveillance, influences tumor progression, immune evasion, metastatic dissemination, and therapeutic response [18]. ccRCC is considered an immunogenic renal cancer subtype characterized by a highly infiltrated TME, with prominent infiltration of immune cell populations, including natural killer (NK) cells, cluster of differentiation (CD) 8+ (CD8^+^) and CD4^+^ T cells, and myeloid cells such as macrophages and neutrophils. Among renal cancer subtypes, ccRCC is distinguished by a particularly pronounced immune-cell infiltration, including substantial CD8^+^ T-cell infiltration, supporting its characterization as an immunologically active tumor subtype [19,20]. To date, the potential role of TME-residing components as immunotherapy biomarkers in RCC has been proposed, including the cytokines interleukin-8 (IL-8) and interleukin-6 (IL-6), as well as the expression of programmed cell death-ligand 1 (PD-L1), as immunotherapy candidates [21,22,23].

Based on ccRCC TME investigation, the therapeutic landscape has changed dramatically over the past decade with the inclusion of immune checkpoint inhibitors, vascular endothelial growth factor (VEGF)-targeted agents, and HIF-2α inhibitors, along with targeted strategies that combine immunotherapy with antiangiogenic therapies, significantly improving survival outcomes and becoming the gold standard for first-line treatment [24,25,26,27]. Unfortunately, at the clinical level, only a minority of patients achieve substantial benefit from these combined therapeutic strategies, while the immune-related adverse events remain a major clinical challenge. In this context, identifying robust immune-related proteomic biomarkers that can serve as stratification criteria for therapeutic administration (to predict therapeutic efficacy, monitor treatment response, and detect toxicity) is essential in personalized oncology. Despite reliable preclinical research and clinical efforts, tissue-based approaches combined with immunohistochemical biomarkers, such as PD-L1 expression and the presence of tumor-infiltrating cytotoxic T lymphocytes, have failed to show consistent predictive utility in ccRCC [28]. However, integrating tissue- and liquid-biopsy-derived proteomic data provides a comprehensive understanding of ccRCC immune biology and enables the development of new biomarker panels for precision oncology. 

This narrative review was conducted using a structured literature search approach to synthesize current knowledge on immune-related proteomic profiling in ccRCC, with a particular focus on tissue and soluble biofluid biomarkers and their implications for immunotherapy, combination treatment strategies, and emerging therapeutic approaches. Literature searches were performed in major scientific databases, including Web of Science, Scopus, PubMed, ScienceDirect, Google Scholar, and ResearchGate, covering publications from 2018 to 2026, while relevant older studies were included when appropriate. The search was conducted between 5 May and 30 July 2026, over the writing period of the review, with an update performed in September 2026. Search terms were combined using the Boolean operators “AND” and “OR” and included various combinations of the following terms: “clear cell renal cell carcinoma,” “renal cell carcinoma,” “proteomics,” “proteomic profiling,” “immune-related biomarkers,” “immune proteomics,” “tumor immune microenvironment,” “tissue biomarkers,” “serum biomarkers,” “plasma biomarkers,” “urine biomarkers,” “soluble biomarkers,” “immunotherapy,” “immune checkpoint inhibitors,” “targeted therapy,” “combination therapy,” “treatment response,” “treatment resistance,” and “emerging therapeutic approaches.” A representative search string was: (“clear cell renal cell carcinoma” OR ccRCC OR “renal cell carcinoma”) AND (proteomics OR “proteomic profiling” OR “immune-related biomarkers” OR “immune proteomics”) AND (tissue OR serum OR plasma OR urine OR “soluble biomarkers” OR immunotherapy OR “immune checkpoint inhibitors” OR “treatment response” OR “treatment resistance”). Search strategies were adapted to the syntax and functionality of individual databases.

Publications were considered eligible if they were peer-reviewed, available in English, and relevant to immune-related proteins or protein-based biomarkers in ccRCC. Eligible evidence included studies investigating tumor tissue, blood, urine, or other soluble biofluids; studies evaluating the diagnostic, prognostic, or predictive value of candidate biomarkers; and clinical or translational investigations addressing associations between protein expression, the tumor immune microenvironment, immunotherapy response, treatment resistance, or therapeutic outcomes. Preclinical studies were considered when they provided mechanistic or translational evidence relevant to the review objectives. Non-peer-reviewed publications, conference abstracts without sufficient experimental data, editorials, opinion articles, and non-English publications were excluded.

Study selection involved an initial assessment of titles and abstracts, followed by full-text evaluation of potentially relevant publications. Reference lists of relevant articles were also examined to identify additional eligible studies. Because this was a narrative review, selection and synthesis were guided by thematic relevance, scientific quality, study design, methodological validity, and the contribution of each study to understanding the clinical or biological significance of candidate biomarkers; no formal meta-analysis or quantitative risk-of-bias assessment was undertaken. The evidence was interpreted according to study type, distinguishing discovery-stage proteomic findings and preclinical observations from findings supported by independent validation cohorts and clinical studies. Particular attention was given to cohort size, analytical methods, validation status, clinical endpoints, and the extent to which reported biomarker associations had been independently replicated. The flow diagram illustrating the literature search and study selection process is presented in Supplementary Figure S1. 

The review is organized thematically as follows. Section 2 and Section 3 address immune-related proteins identified in ccRCC tissue and soluble biofluids, respectively. Section 4 examines immunotherapy and potential predictive biomarkers, while Section 5 discusses combination treatment strategies and emerging therapeutic approaches. The final section, Section 6 considers current limitations, future research priorities, and clinical perspectives.

## 2. Immune-Related Tissue Proteins as Biomarkers in ccRCC

Among the numerous biomarkers investigated, immune checkpoint proteins have attracted increasing interest because of their close association with tumor progression and immune regulation [29]. Although several checkpoint proteins have demonstrated promising prognostic, predictive, and diagnostic potential, their clinical application has been limited by conflicting evidence, together with the intratumoral heterogeneity of ccRCC [30]. Beyond T-lymphocyte-associated protein 4 (CTLA-4) and the programmed cell death protein 1 (PD-1)/PD-L1 axis, growing attention has been raised to other immune checkpoint proteins with potential prognostic value in ccRCC. Among them, T-cell immunoglobulin and mucin-domain containing-3 (TIM-3), lymphocyte-activation gene 3 (LAG-3), and indoleamine 2,3-dioxygenase 1 (IDO-1) have attracted significant interest because of their roles in immune regulation and tumor progression [31]. Although evidence remains scarce, these molecules, along with others reported below, have shown promising prognostic, predictive, and diagnostic potential and could represent future therapeutic targets [32]. The following section summarizes the main immune-related tissue protein biomarkers studied in ccRCC and their clinical significance.

### 2.1. PD-1/PD-L1/PD-L2 Axis

#### 2.1.1. PD-L1 (Programmed Death-Ligand 1) as a Prognostic Biomarker

Regarding immune checkpoint proteins, the cell-surface receptor PD-1 and its ligands, PD-L1 and programmed death-ligand 2 (PD-L2), have been extensively investigated as prognostic and predictive biomarkers in ccRCC. Specifically, PD-L1 is the most extensively studied immune checkpoint biomarker in this malignancy [30]. Several studies have demonstrated that PD-L1 expression is associated with poor prognosis and less favorable clinical outcomes in patients with ccRCC [33,34]. Increased PD-L1 expression is frequently correlated with advanced tumor grade, progressed T stage, and an elevated metastatic risk in distant organs. Furthermore, tumors expressing PD-L1 have been reported to be associated with poor survival outcomes and a higher risk for disease progression [35]. A ccRCC cohort study, evaluating PD-L1 protein expression in tumor tissue by immunohistochemistry (IHC), found PD-L1 positivity in 20.9% of participants, which was associated with decreased 5-year OS (46.7% vs. 66.0% in PD-L1-negative tumors; hazard ratio [HR] 2, 95% confidence interval [CI] 1.2–3.3, *p* = 0.005) [36]. Collectively, these findings support PD-L1 as an adverse prognostic biomarker linked to poor patient outcomes in ccRCC [30].

#### 2.1.2. PD-L1 as a Predictive Biomarker for Immunotherapy

Although PD-L1 expression is established as a predictive biomarker for immunotherapy in several malignancies, its predictive value for stratifying ccRCC patients undergoing immunotherapy remains unproven [30]. This is mainly due to conflicting evidence regarding the association between PD-L1 protein levels and response to immune checkpoint inhibitors (ICIs) [33]. Exploratory analyses from the phase III CheckMate 214 trial (NCT02231749) suggested a greater clinical benefit with nivolumab plus ipilimumab in patients with PD-L1 expression ≥ 1% (assessed through IHC), although an OS benefit was also observed in patients with PD-L1 expression < 1% [37]. In contrast, the phase III CheckMate 025 trial (NCT01668784) demonstrated that nivolumab, a monoclonal antibody targeting PD-1, improved outcomes regardless of PD-L1 expression status. However, patients with PD-L1-positive tumors had shorter OS across treatment groups, supporting that PD-L1 expression was associated with a poor prognosis rather than with improved response to nivolumab [33]. Furthermore, clinical benefit has also been observed in a considerable subset of patients with PD-L1-negative tumors, whereas many PD-L1-positive patients fail to respond to treatment. Collectively, these findings indicate that PD-L1 protein expression alone is not sufficient to accurately predict response to ICIs in patients with ccRCC and, therefore, cannot currently be considered a reliable stand-alone predictive biomarker [34].

#### 2.1.3. Limitations of PD-L1 Clinical Application 

Despite its prognostic and predictive potential in ccRCC, clinical application of PD-L1 as a biomarker remains challenging [33]. Several factors limit its clinical use for guiding treatment selection with ICIs. Among them, PD-L1 expression can be induced by multiple mechanisms, including oncogenic signaling pathways, hypoxia, and interferon-γ (IFN-γ)-mediated immune responses [30]. Furthermore, its predictive value remains controversial due to methodological heterogeneity across studies, including differences in detection methods, antibody clones, detection thresholds, and scoring algorithms [33]. The marked intratumoral heterogeneity of ccRCC, combined with the discordance in PD-L1 expression between primary and metastatic lesions, further limits PD-L1 reliability as a predictive biomarker [33].

#### 2.1.4. PD-1 (Programmed Death 1) as Prognostic Biomarker

Compared with PD-L1, the prognostic significance of PD-1 in ccRCC has been examined in a limited number of studies, primarily focusing on its protein expression assessed by IHC in tumor tissue samples. PD-1 expression is mainly detected in tumor-infiltrating mononuclear cells (TIMCs), particularly tumor-infiltrating lymphocytes (TILs), and it has been associated with adverse clinicopathological characteristics, including advanced tumor grade, progressed tumor-node-metastasis (TNM) stage, sarcomatoid differentiation, and shortened survival time [35,38]. Interestingly, Granier et al. reported that co-expression of PD-1 with TIM-3 increased the prognostic significance of the former, suggesting that the combined evaluation of multiple immune checkpoint proteins may provide greater prognostic value than PD-1 expression alone [39]. In another study, PD-1 positive cells were associated with an increased risk of distant metastatic relapse (DMR) (HR 6.146, 95% CI, 1.249–30.238, *p* = 0.026) and shorter OS (HR 3.195, 95% CI 1.046–9.761, *p* = 0.042) [40]. However, the available evidence remains inconsistent, as some studies have failed to demonstrate an independent association between PD-1 expression and patient survival, emphasizing the need for intensifying prospective validation [35].

#### 2.1.5. PD-L2 (Programmed Death-Ligand 2)

Only a few studies have explored the clinical significance of PD-L2 in ccRCC. Increased PD-L2 expression has been linked to lymph node metastasis and reduced cancer-specific survival in patients with ccRCC, suggesting its potential prognostic role [35,41]. In addition, detection of PD-L2 at the protein level by IHC in tumor tissue samples, has been reported to correlate with PD-L1 expression, although PD-L2 can also be detected independently of PD-L1 in certain tumors [42]. Moreover, a subgroup analysis in ccRCC showed that elevated PD-L2 expression was linked to poorer disease-free survival/progression-free survival (DFS/PFS) (HR 1.45, 95% CI = 1.03–2.03, *p* = 0.033) [43]. The available evidence remains limited, and the clinical significance of PD-L2 has not yet been fully established. Interestingly, in chRCC, protein expression of PD-L2 rather than PD-L1 has been associated with decreased OS, suggesting that the prognostic value of immune checkpoint proteins may vary between RCC histological subtypes [38,42].

### 2.2. CTLA-4 (Cytotoxic T-Lymphocyte Associated Protein 4)

It is well established that CTLA-4 plays a crucial role in the regulation of T-cell responses [44]. The prognostic significance of CTLA-4 as a biomarker has also been investigated in ccRCC at both protein and gene expression levels, through immunohistochemical analysis of tumor tissue samples and transcriptomic data approaches, respectively [33]. Several malignancies have been reported to show increased CTLA-4 gene expression, including esophageal carcinoma, invasive breast carcinoma, and ccRCC. In addition, polymorphisms at the *CTLA-4* locus have been linked to an elevated risk of developing high-stage ccRCC [33,45]. CTLA-4 expression on TIMCs has been detected more frequently in patients with primary metastatic disease, with increased expression in advanced tumor stages [45]. Another study showed that elevated CTLA-4 expression was associated with reduced OS in patients with ccRCC, irrespective of metastatic status [33,46]. Multivariate analysis identified CTLA-4 expression as an independent prognostic biomarker for ccRCC, with its expression being related to aggressive clinicopathological characteristics and poor clinical outcome [45]. Furthermore, a computed tomography-based radiomics model showed that increased CTLA-4 expression was associated with poor OS (HR 1.458, 95% CI 1.13–1.881, *p* = 0.004) [47]. Notably, a statistically significant positive correlation has been reported between PD-1 and CTLA-4 expression, and their co-expression has been correlated with higher tumor stage, higher tumor grade, shorter OS, and adverse clinical outcomes [45,46].

### 2.3. TIM-3 (T-Cell Immunoglobulin and Mucin-Domain Containing-3)

Among the emerging immune checkpoint proteins, TIM-3 is the most extensively investigated biomarker for ccRCC, with its expression investigated at the protein level in tumor tissue samples [31]. TIM-3 is frequently co-expressed with other immune checkpoint proteins, including PD-1, LAG-3, and T-cell immunoreceptor with immunoglobulin and ITIM domains (TIGIT), on tumor-infiltrating CD8+ T cells, contributing to T-cell exhaustion and the establishment of an immunosuppressive tumor microenvironment [39,41]. Several studies have reported that increased TIM-3 expression is associated with advanced tumor stage and shorter OS and PFS in ccRCC patients [48]. For instance, in a study of 91 patients, the TIM-3-positive cancer cells were present in 69.2% of the tumors and were associated with shorter PFS [49]. Co-expression of TIM-3 and PD-1 has been linked to a more aggressive tumor phenotype and unfavorable clinical outcome [39]. These findings suggest that TIM-3 may represent a promising prognostic biomarker, providing an exploratory basis for evaluating the combined TIM-3/PD-1 blockade in future therapeutic strategies [39].

### 2.4. LAG-3 (Lymphocyte-Activating Gene 3)

Similar to TIM-3, LAG-3 has also emerged as a potential biomarker in ccRCC [32]. LAG-3 is another cell-surface receptor that is present on T cells and NK cells and acts as an inhibitory regulator of T-cell activity [50]. LAG-3 is frequently co-expressed with PD-1 and has been associated with poor prognosis and reduced OS in patients with RCC. Liu et al. demonstrated that based on transcriptomic analysis high mRNA expression of LAG-3 was linked to poorer OS, PFS, and disease-specific survival (DSS) (HR 1.236, 1.121, 1.269, respectively, with *p* < 0.005 [51]. Although its predictive value remains to be established, LAG-3 is currently being evaluated as a potential therapeutic target, particularly in combination with PD-1 blockade [31]. 

### 2.5. IDO-1 (Indoleamine 2,3-Dioxygenase 1)

Another emerging biomarker is IDO-1, an immunoregulatory enzyme involved in tumor immunosurveillance escape. Increased IDO-1 protein expression has been associated with shortening of OS time in patients with RCC, supporting its potential role as a prognostic biomarker [41]. In addition, IDO-1’s immunosuppressive function has led to its investigation as a promising therapeutic target [44]. Consequently, several studies are currently evaluating strategies combining IDO-1 inhibition with immune checkpoint blockade, aiming to improve clinical outcomes in patients with advanced ccRCC [41].

Overall, immune checkpoint proteins represent a promising group of prognostic and predictive biomarkers in ccRCC, although their clinical utility remains incompletely established. Among the best-studied markers, PD-L1, PD-1, PD-L2, and CTLA-4 have shown potential prognostic significance, particularly when assessed in combination with other immune checkpoint molecules. Emerging biomarkers, including TIM-3, LAG-3, and IDO-1, highlight the complexity of the immune landscape in ccRCC and may provide additional prognostic information and therapeutic opportunities. Overall, ccRCC heterogeneity, differences in biomarker assessment methods, and the frequent co-expression of multiple immune checkpoints underscore the need for standardized evaluation and prospective validation. Future research should therefore focus on developing multidimensional biomarker strategies that integrate several immune checkpoint proteins with clinicopathological and molecular features to improve patient stratification and guide personalized immunotherapy.

### 2.6. Other Immune-Related Tissue Protein Biomarkers

Except for the immune checkpoint proteins discussed above, recent studies have identified additional immune-related molecules with promising prognostic significance in ccRCC. These candidates remain under exploratory investigation, yet preliminary results suggest they may improve prognostic assessment of disease progression [29].

Deoxyribonuclease 1-like 3 (DNASE1L3) has recently emerged as a potential tumor-suppressive biomarker in ccRCC. DNASE1L3 expression has been investigated at mRNA levels using transcriptomic analysis of renal tissue samples and at protein levels based on Western blot analysis of renal cell lines. Growing evidence suggests that DNASE1L3 plays an important role in ccRCC progression, and that its overexpression suppresses aggressive tumor cell behavior. Compared with normal renal tissue, DNASE1L3 mRNA expression is markedly reduced in tumor samples (7.98 vs. 10.87, *p* < 0.001) and is associated with tumor size (r = −0.32, *p* < 0.001), tumor weight, clinical stage, and histological grade according to RNA-seq data. Functional experiments showed that DNASE1L3 overexpression suppresses tumor cell proliferation and invasion, possibly by inhibiting the phosphoinositide 3-kinase/protein kinase B (PI3K/AKT) signaling pathway, supporting its potential as both a prognostic biomarker and a therapeutic candidate [52].

Complement C1q C chain (C1QC) has attracted attention as a potential immune-related biomarker in ccRCC. Yao et al. investigated C1QC expression at both mRNA and protein levels through transcriptomic analysis and based on the immunohistochemical data. Elevated mRNA expression of C1QC has been linked to advanced tumor stage, higher tumor grade, lymph node metastasis, and shorter OS time (HR 1.67, 95% CI 1.23–2.27, *p* = 0.00097). C1QC knockdown suppresses tumor proliferation, migration, and invasion, supporting its involvement in ccRCC progression. Moreover, C1QC expression strongly correlates with immune cell infiltration, particularly macrophages, suggesting it may contribute to tumor progression by modulating the tumor immune microenvironment [53].

Similar observations have been reported for mitotic spindle organizing protein 2A (MZT2A), whose gene expression has been investigated as a potential biomarker in ccRCC. Increased MZT2A expression has been connected to advanced disease stage and pathological grade, while patients with elevated MZT2A levels exhibit significantly shorter OS (*p* < 0.05). Notably, MZT2A expression has been correlated with multiple immune regulators and immune cell infiltration, indicating its possible involvement in the tumor immune microenvironment. These findings support the potential role of MZT2A as both a diagnostic and prognostic biomarker in ccRCC [54].

For ccRCC management, vimentin has emerged as another candidate prognostic marker. Increased vimentin protein expression, assessed by IHC in tumor tissue samples, has been linked to clinicopathological characteristics including vascular invasion (odds ratio [OR] 2.90, 95% CI 1.05–7.62, *p* = 0.031), distant metastatic spread (OR 3.10, 95% CI 1.01–12.54, *p* = 0.048), and greater tumor size (*p* = 0.004). These associations suggest that vimentin expression may reflect tumor aggressiveness and disease progression, while its correlation with poor survival further supports its potential contribution to prognostic assessment in ccRCC. Additional validation is required before its use in routine clinical practice [55].

A panel of angiogenesis-related genes identified through transcriptome analysis has been proposed as a prognostic signature in ccRCC because of its ability to distinguish patients with different survival outcomes. Among the genes of this panel, runt-related transcription factor 1 (*RUNX1*) showed the strongest prognostic contribution. Further analyses showed that *RUNX1* may also regulate immune cell infiltration and lipid metabolic pathways, suggesting a crucial role in ccRCC biology beyond angiogenesis. Experimental analyses further supported its biological significance: overexpression promoted RCC cell proliferation and migration in vitro, and in vivo mouse models showed enhanced tumor growth [56]. Another study revealed that patients with increased protein expression of RUNX1 had poorer clinical survival, and RUNX1 was also identified as an independent marker of poor prognosis (HR 1.58, 95% CI 1.054–2.371, *p* = 0.027) after adjustment for age, tumor stage, necrosis, and grade [57].

Carbonic anhydrase IX (CAIX) has also been extensively investigated as a biomarker in ccRCC because of its high protein expression on the surface of tumor cells in this subtype and its established use in immunohistochemical evaluation. High CAIX expression has been associated with lower tumor stage and improved survival outcomes, suggesting a potential prognostic role in ccRCC. However, beyond immunohistochemistry, its applications remain exploratory and require further clinical validation [58].

As a well-established marker of cellular proliferation with a crucial role in mitosis, Ki-67 has achieved greater prominence for its potential prognostic value in RCC. In non-metastatic ccRCC with tumor thrombus, high protein expression of Ki-67 was correlated with shorter survival, together with adverse pathological features, including sarcomatoid differentiation, and is indicative of a more aggressive tumor phenotype. In this study, a Ki-67 cut-off of 30% best distinguished patients with different survival outcomes, and 21.3% of participants showed high Ki-67 expression with worse recurrence-free survival (RFS) and cancer-specific survival (CSS) (all *p* < 0.001). However, this study has significant limitations, and further validation is required [59]. 

Protein levels of cadherin examined by IHC in tumor tissue samples are associated with tumor aggressiveness. According to the reported findings, decreased E-cadherin levels were frequently observed in tumors with adverse clinicopathologic characteristics, including lymph node metastasis and increased tumor size. In contrast, high P-cadherin and N-cadherin expression were correlated with higher nuclear grade and advanced stage. Changes in E-cadherin expression were also linked to worse OS and may provide useful prognostic information in ccRCC in the future [60].

Collectively, these candidates represent promising biomarkers in ccRCC and may further improve prognostic assessment. However, validation in large prospective cohorts is still required prior to their application in the clinical routine. Table 1 summarizes the biological characteristics and clinical relevance of the immune checkpoint-related and other immune-related biomarkers discussed above.

## 3. Immune-Related Biofluid Proteins as Potential Biomarkers in ccRCC

The tissue-based proteins discussed above represent localized biomarkers, but their clinical assessment generally requires invasive procedures. In contrast, liquid biopsy provides a minimally invasive approach for detecting diagnostic, prognostic, and predictive biomarkers in circulating biofluids. Shifting from localized tissue profiles to circulating or urinary proteomes enables broader disease assessment, since soluble proteins are generally more accessible biomarkers for early detection, monitoring, and management of ccRCC [61]. These biomarkers have been explored for several distinct clinical purposes, including disease detection, prognostic stratification, monitoring of disease progression, and assessment of treatment response. However, the evidence supporting each application varies considerably among individual proteins, and associations with disease severity or survival do not necessarily establish diagnostic accuracy or treatment-specific predictive value. This section therefore distinguishes diagnostic, prognostic, and potential predictive applications of circulating biomarkers, considering the biological material analyzed, the methods and thresholds used for their assessment, and the extent of independent validation where reported.

**Figure 1:**
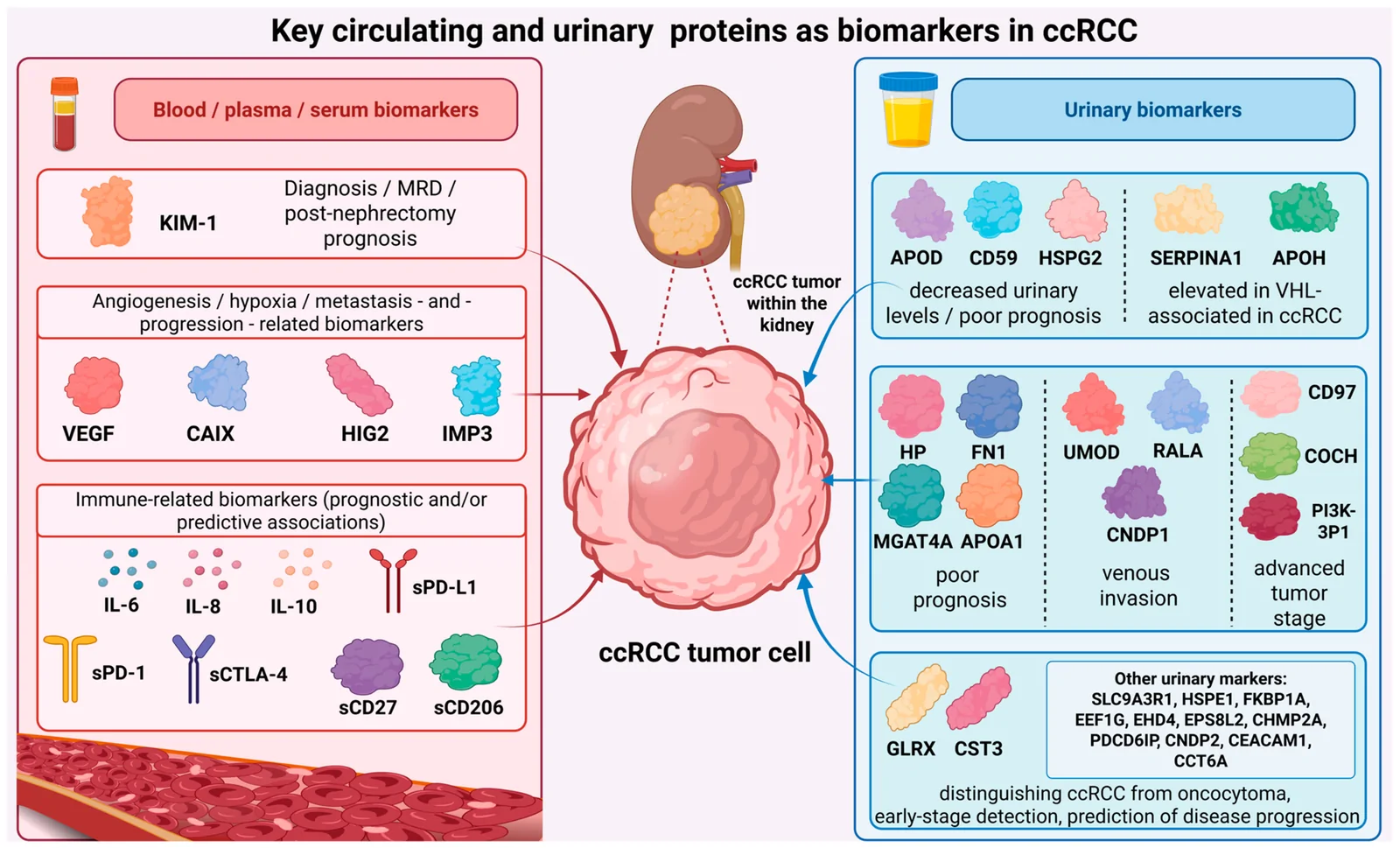
Key circulating and urinary protein biomarkers in clear cell renal cell carcinoma (ccRCC). The figure illustrates selected biomarkers detected in blood, plasma, serum and urine that have been associated with ccRCC, including diagnosis, prognosis, metastatic progression and immune-related changes. Circulating biomarkers are shown on the left and urinary biomarkers are presented on the right. Created in BioRender. Anatoliotaki, C. (2026). https://BioRender.com/0db6p55. Abbreviations: APOA1, apolipoprotein A1; APOD, apolipoprotein D; APOH, apolipoprotein H; CAIX, carbonic anhydrase IX; CCT6A, chaperonin containing TCP1 subunit 6A; CD59, cluster of differentiation 59; CD97, cluster of differentiation 97; CEACAM1, CEA cell adhesion molecule 1; CHMP2A, charged multivesicular body protein 2A; CNDP1, carnosine dipeptidase 1; CNDP2, carnosine dipeptidase 2; COCH, cochlin; CST3, cystatin C; EEF1G, eukaryotic translation elongation factor 1 gamma; EHD4, EH domain containing 4; EPS8L2, EPS8 like 2; FKBP1A, FKBP prolyl isomerase 1A; FN1, fibronectin 1; GLRX, glutaredoxin; HIG2, hypoxia-inducible protein 2; HP, haptoglobin; HSPE1, heat shock protein family E (Hsp10) member 1; HSPG2, heparan sulfate proteoglycan 2; IL-6, interleukin-6; IL-8, interleukin-8; IL-10, interleukin-10; IMP3, insulin-like growth factor II mRNA-binding protein 3; KIM-1, kidney injury molecule 1; MGAT4A, alpha-1,3-mannosyl-glycoprotein 4-beta-N-acetylglucosaminyltransferase A; MRD, minimal residual disease; PDCD6IP, programmed cell death 6 interacting protein; PIK3IP1, phosphoinositide-3-kinase interacting protein 1; RALA, RAS like proto-oncogene A; sCD27, soluble cluster of differentiation 27; sCD206, soluble cluster of differentiation 206; sCTLA-4, soluble cytotoxic T-lymphocyte-associated protein 4; SERPINA1, serpin family A member 1; SLC9A3R1, solute carrier family 9 member A3 regulator 1; sPD-1, soluble programmed cell death protein 1; sPD-L1, soluble programmed cell death-ligand 1; UMOD, uromodulin; and VEGF, vascular endothelial growth factor.

In 2023, Liu et al. studied early-stage ccRCC to investigate proteomic relationships among tissue, plasma, and urine. Their results suggest that proteomic profiling in biofluids reflects not only tumor-driven functional changes but also host immune responses, offering a viable, non-invasive alternative to tissue biopsies. Specifically, proteomic profiling revealed differential abundance of cytoskeletal proteins and metabolic enzymes in plasma, whereas urinary proteomes showed differential levels of proteins involved in cell adhesion and host defense. In fact, fibroblast growth factor receptor 1 (FGFR1), glutamic-oxaloacetic transaminase 1 (GOT1), and fibroblast growth factor binding protein 2 (FGFBP2) demonstrated strong diagnostic performance for ccRCC (AUC of 0.981, with 96.3% sensitivity and 92.6% specificity. Similarly, cholesteryl ester transfer protein (CETP), seizure-related 6 homolog like 2 (SEZ6L2), and cytochrome c oxidase subunit 5B (COX5B) detection in urine showed an AUC of 0.970, with 92.6% sensitivity and 92.6% specificity, underscoring the potential diagnostic value of these protein panels in ccRCC [62]. The following section summarizes the main immune-related soluble protein biomarkers studied in ccRCC and their possible clinical significance. Figure 1 illustrates key circulating and urinary protein biomarkers investigated in ccRCC, including immune-related and other soluble proteins with promising diagnostic, prognostic, and predictive significance.

### 3.1. Circulating Blood (Plasma/Serum) Proteins

#### 3.1.1. cKIM-1 

A variety of soluble circulating proteins has been investigated in the past decades with the kidney-injury molecule-1 (KIM-1), a transmembrane protein highly expressed in T-cells and epithelial cells of ccRCC, and has recently gained attention. Detection of KIM1 in circulating fluids has been associated with an 83% specificity of diagnosing early-stage tumors, which increases to 97% in advanced stages [63]. In fact, high-grade ccRCC tumors exhibited approximately a seven-fold increase in KIM1 protein abundance, whereas patients with metastatic ccRCC (mccRCC) had significantly elevated concentrations of KIM1 in the plasma [63]. The ASSURE clinical trial, performed in patients, the majority of which had ccRCC (79%), evaluated administration of adjuvant receptor tyrosine kinase (RTK) inhibitors such as sunitinib along with sorafenib in respected high-risk RCC patients. Detection of KIM-1 in the plasma of patients that underwent higher post-nephrectomy was associated with worse DFS and OS (survival time ratio for DFS: HR 0.56, 95% CI 0.42–0.73, *p* < 0.001), indicating that KIM-1 is a potential biomarker for minimal residual disease (MRD) [64]. In agreement with these results, another study reported that increased post-nephrectomy levels of circulating KIM-1 in serum of patients with ccRCC or sarcomatoid components was linked also to worse DFS (median DFS: 35.9 vs. 57.2 months for KIM-1-high vs. KIM-1-low; HR 1.75, 95% CI 1.40–2.17) but improved clinical outcomes with atezolizumab (immunotherapy) vs. placebo (HR 0.72, 95% CI 0.53–0.99) [65]. Interestingly, a novel analysis of plasma KIM-1 in oligometastatic ccRCC showed that shorter systemic therapy-free survival (STFS) was associated with high KIM-1 at baseline (HR: 2.45, 95% CI 1.48–4.08; *p* < 0.001), revealing its prognostic value as a biomarker and suggesting a prognostic model (K-COMPASS) for STFS. However, compared to circulating tumor DNA (ctDNA), KIM-1 was significantly associated with PFS and OS, indicating that the two biomarkers could signify different aspects of tumor biology [66]. 

Overall, circulating KIM-1 has been investigated as a diagnostic candidate and a prognostic biomarker in ccRCC, particularly for postoperative recurrence risk and subsequent disease outcomes. Its potential predictive relevance requires more careful interpretation, as associations between baseline or postoperative KIM-1 concentrations and outcomes during a particular treatment do not, in isolation, establish that the marker identifies differential treatment benefit. The diagnostic performance and prognostic associations reported to date should be interpreted in relation to the study population, specimen type, assay, sampling time, and cutoff used. Independent validation and standardized assessment are needed to determine the clinical applicability of circulating KIM-1 across disease stages and treatment settings.

#### 3.1.2. cVEGF

Soluble proteins such as VEGF, IL-6, IL-8, and interleukin-10 (IL-10) are key drivers of carcinogenesis and myeloid inflammation and consequently promote an immunosuppressive TME. Serum levels of VEGF prior to nephrectomy have been shown to predict post-operative recurrence in nonmetastatic ccRCC with the recurrence-free survival rate in patients with a high serum VEGF level (>416 pg/mL) to be significantly lower than in patients with a low serum VEGF level [67]. Great emphasis has been placed on baseline circulating soluble VEGF levels, which have been associated with poor survival outcomes in patients receiving first-line nivolumab–ipilimumab therapy (BIONIKK), pretreated patients with mRCC undergoing nivolumab immunotherapy (NIVOREN), and patients with advanced ccRCC treated with the anti-angiogenic agent sorafenib [68,69,70]. In contrast, in advanced ccRCC patients treated with pembrolizumab–axitinib, baseline serum concentrations of VEGF and vascular endothelial growth factor receptor 2 (VEGFR2) showed no significant correlation with clinical outcome [71]. In the JAVELIN Renal 101 clinical trial, high levels of circulating VEGF were associated with shorter PFS specifically in patients receiving sunitinib, acting as a biomarker of resistance to standard anti-angiogenic therapy [72]. Remarkably, in a retrospective study of patients who received a gallium-68 prostate-specific membrane antigen positron emission tomography/computed tomography (68Ga-PSMA PET/CT) scan for diagnosing and staging ccRCC, circulating VEGF levels over 67.71 pg/mL correlated with 85% accuracy in predicting bright tumor signals on PSMA scans. This, combined with low tumor volume rate and post-surgery VEGF monitoring, may be a highly specific method to track and predict tumor prognosis risk [73]. 

Taken together, circulating VEGF has shown potential prognostic value in ccRCC, including associations with postoperative recurrence and survival in selected treatment cohorts. Its potential predictive role appears to depend on the therapeutic context and remains insufficiently established for routine treatment selection. The differing findings across treatment regimens underscore the need to distinguish general prognostic associations from evidence that VEGF levels modify the relative benefit of a specific therapy. The reported concentration thresholds should be considered study-specific unless independently validated in comparable populations.

#### 3.1.3. Circulating Interleukins (cIL): IL-6, IL-8, and IL-10 

M2 macrophages secrete the immunosuppressive mediator IL-10 and express the inhibitory molecule PD-L1, and they are largely responsible for suppressing the anti-tumor immune response in the renal tumor TME. Moreover, IL-6 and IL-8 inhibit the cytotoxic T-cell response by driving myeloid-derived suppressor cell infiltration into the tumor TME. All three proteins have been associated with poor clinical outcome in several tumors, including ccRCC [74,75,76].

#### 3.1.4. cIL-6

In ccRCC, circulating IL-6 levels are strongly linked to tumor immunity. High levels of serum IL-6 serve as a negative predictor (poor clinical outcome) for first-line pembrolizumab/axitinib treatment (PFS: HR: 3.51, *p* = 0.003; OS: HR: 7.18, *p* = 0.001), suggesting that serum IL-6 could be used as a non-invasive predictive biomarker to guide advanced RCC treatment selection, benefiting patients from alternative therapeutic strategies [77]. Although the predictive value of IL-6 under sunitinib as a first-line treatment for metastatic RCC (mRCC) has been evaluated, few studies enrolled patients with ccRCC or a profile of poor International Metastatic Renal Cell Carcinoma Database Consortium (IMDC) risk [78,79]. At baseline, serum IL-6 levels contribute to metastatic status diagnosis, while the combination of IL-6 and interleukin-27 (IL-27) significantly predicted patient survival and tumor recurrence [80]. Yet, the prognostic value of this cytokine is underscored by data from the NIVOREN study, where high levels of circulating IL-6 were negatively associated with PFS and OS in nivolumab-treated advanced RCC patients (PFS: HR = 1.68, *p* = 0.0021; OS: HR = 3.28, *p* < 0.0001) [68,81]. Similarly, ccRCC patients with undetectable serum IL-6 had longer PFS than those with detectable IL-6 (HR = 0.4; *p* = 0.028) [71]. Nevertheless, a recent meta-analysis of ccRCC patients showed that high IL-6 levels correlated with significantly poorer OS and PFS, suggesting that circulating IL-6 is a promising prognostic biomarker in RCC [82].

#### 3.1.5. cIL-8

Although C-X-C motif chemokine ligand 8 (CXCL8), also known as IL-8, is one of the most extensively studied chemokines in solid tumors, evidence for its role as a biomarker specific to ccRCC remains limited. A recent analysis across multiple immune checkpoint inhibitor trials for various malignancies, including mRCC, demonstrated that serum IL-8 acts as a dual-purpose biomarker. It serves as a negative prognostic marker across solid tumors while simultaneously acting as a predictive biomarker of ICI resistance, thereby facilitating precise monitoring of therapeutic response [83]. In RCC patients treated with single-agent everolimus (targeted therapy) instead of ICIs, elevated baseline serum IL-8 levels were associated with reduced OS in non-immunotherapy trial arms, indicating the high prognostic value of serum IL-8 [76]. Similarly, current data from the phase 2 NIVOREN study showed that high circulating IL-8 levels correlate with poor outcomes in patients with advanced RCC pretreated with nivolumab (HR = 2.57, *p* < 0.001). These elevated IL-8 levels were also linked to the myeloid gene expression signature identified in the IMmotion150 trial (*p* = 0.041) [68,81]. Interestingly, a meta-analysis evaluating patients with ccRCC confirmed that high levels of circulating IL-8 were significantly correlated with poor prognosis in RCC, since elevated IL-8 was associated with shortening of PFS and OS, respectively ([HR]: 1.27, 95% confidence interval [CI]: 1.01–1.59; *p* = 0.037; HR: 1.85, 95% CI: 1.21–2.84; *p* = 0.001). Revised data proposed integrating soluble cytokines, mainly IL-6, IL-8, and selected chemokines such as C-X-C motif chemokine ligand 10 (CXCL10), with signal transducer and activator of transcription 3 (STAT3) activation to enhance diagnostic and prognostic precision in ccRCC [84]. These results suggest that IL-8 could be a valuable protein marker for RCC prognosis [85].

Other evidence shows contradictory results for circulating interleukins. Even though preoperative soluble IL-10 was detectable in only 13.2% of the ccRCC cohort [86], conversely, elevated baseline levels of IL-10 in a different ccRCC cohort were associated with poor responses to first-line ICI therapy, indicating its value as a predictive marker in advanced RCC rather than a prognostic marker as is commonly suggested [87]. Notably, in patients with metastatic RCC, high serum levels of soluble IL-10 were detected; however, no association between high serum levels and adverse prognostic outcomes was observed [88,89].

Overall, circulating IL-6 and IL-8 have shown relatively consistent associations with adverse clinical outcomes in RCC, supporting their investigation as prognostic biomarkers. Their potential predictive relevance during immune checkpoint inhibitor therapy remains dependent on treatment context and requires validation beyond associations observed within individual treatment cohorts. Evidence for circulating IL-10 is more limited and inconsistent, with reported associations involving both adverse treatment response and the absence of a clear relationship with prognosis. Accordingly, these cytokines should be regarded as candidate biomarkers rather than established stand-alone tools for selecting immunotherapy or guiding treatment changes.

#### 3.1.6. Circulating PD-L1 and PD-1

High circulating levels of soluble PD-L1 (sPD-L1) have been identified as a significant indicator of tumor progression, with RCC patients presenting metastatic disease at diagnosis and reduced cancer-specific survival [86]. In patients with mRCC, high baseline levels of circulating sPD-L1 have been negatively associated with survival in treatment-naïve patients receiving sunitinib, further supporting the role of soluble PD-L1 and PD-1 as intrinsic adverse prognostic biomarkers [90]. Additionally, assessment of sPD-L1 levels in patients with mRCC undergoing nivolumab therapy indicated that this marker may predict treatment response [91]. By distinguishing responders from non-responders at baseline, plasma soluble PD-1 (sPD-1), sPD-L1, and soluble butyrophilin subfamily 3 member A1 (sBTN3A1) were shown to be promising predictors of response to nivolumab in patients with mRCC, demonstrating an improved PFS. Indeed, measuring sPD-1, sPD-L1, and sBTN3A1 offers a non-invasive, real-time monitoring strategy to evaluate dynamic changes during RCC progression and treatment [92].

However, research on sPD-1 and sPD-L1 has primarily focused on patients with non-metastatic ccRCC prior to surgery, demonstrating associations with poor prognosis, larger tumor size, and higher tumor grade, respectively [93,94,95]. In a cohort of RCC patients treated with nivolumab, both high baseline sPD-L1 levels and an increase in sPD-L1 during treatment were associated with disease progression [96]. These findings are consistent with those reported by Sokolov et al., who demonstrated that median sPD-1 and sPD-L1 levels were significantly higher in patients with RCC than in healthy controls [97]. In a subsequent study published the same year, the same group evaluated a cohort of 90 patients with ccRCC and found that elevated soluble PD-1 and PD-L1 levels were associated with worse OS. Specifically, patients with an sPD-L1 concentration ≥ 42.6 pg/mL exhibited a significantly lower 5-year OS compared to cohorts with lower concentrations (*p* = 0.0012), highlighting the potential of sPD-L1 as a biomarker for patient stratification [97]. In another study evaluating circulating sPD-1 and sPD-L1 levels, the investigators found no correlation with basic clinicopathological parameters, even though both markers were significantly elevated in patients with mccRCC. Nevertheless, sPD-1 was specifically associated with IMDC risk groups and Morphology, Attenuation, Size, and Structure (MASS) response criteria during systemic therapy. Furthermore, the authors demonstrated that tumor PD-L1 immunostaining and high plasma sPD-L1 concentrations (>793 ng/mL) were independently associated with poor survival. They concluded that combining tumor tissue PD-L1 expression with plasma sPD-L1 positivity substantially improves prognostic accuracy, underscoring the complementary value of soluble and immunohistochemical PD-L1 as clinical biomarkers in ccRCC [98]. Notably, Wang et al. reported that soluble PD-L2 (sPD-L2), but not sPD-L1, was predictive of recurrence risk in ccRCC, and that sPD-L2 was expressed at higher levels compared to sPD-L1, which is consistent with the findings of Giraldo and colleagues [95,99]. Potential sources of sPD-L2 include tumor-derived exosomes [100] and alternatively activated macrophages [101], both of which can inhibit T cell-mediated antitumor immune responses. Thus, the independent role of sPD-L1 in ccRCC clinical management remains controversial.

Circulating soluble PD-1 and PD-L1 have been investigated primarily as prognostic candidates, with additional exploratory evidence relating baseline concentrations or on-treatment changes to response and disease progression during immunotherapy. These findings should be distinguished from the predictive role investigated for tumor PD-L1 immunostaining, as soluble and tissue-associated proteins represent different biological measurements. Reported concentration thresholds and associations vary across studies, and their clinical interpretation depends on the assay, specimen, sampling time, and patient population. Although combined assessment of tissue and circulating markers may improve risk stratification, independent validation is required before these approaches can be considered reliable clinical decision tools. Evidence for soluble PD-L2 should likewise be described separately, particularly where the reported outcome concerns recurrence risk rather than treatment-specific benefit.

#### 3.1.7. cCDs: CD27, CD80, CD137, CD28 (And CTLA-4), CD163, CD206

In the ccRCC TME, various clusters of differentiation (CD) molecules have been shown to play crucial roles in shaping the TME, regulating immune responses, and influencing disease progression, prognosis, and response to therapy [102,103]. Given the limitations of tissue-based assessment, there is growing interest in circulating soluble CD proteins relevant to RCC as minimally invasive biomarkers. These novel biomarkers may provide real-time insights into systemic immune alterations and facilitate more effective management of ccRCC. 

Among solid tumors, ccRCC expresses the highest levels of CD70, a co-stimulatory ligand that activates CD27-expressing T cells. This interaction results in the release of soluble CD27 (sCD27), supporting its potential role as a circulating biomarker in patients with CD70-positive ccRCC [104,105,106]. High plasma concentrations of soluble CD27 have been reported to correlate with intratumoral CD27–CD70 interactions. Soluble CD27 also associates with poor OS specifically in patients with ccRCC receiving anti-PD-1 therapy but not in patients treated with antiangiogenic agents (HR: 5.02, *p* = 0.004). These findings suggest that soluble CD27, as a marker of T-cell dysfunction, may serve as a predictive biomarker of resistance to immunotherapy in ccRCC [107]. In 2019, Wang et al. profiled circulating levels of 14 immune checkpoint-related proteins in 182 patients with ccRCC, including CD28, CD80, CD137, CD27, and CTLA-4, and investigated their associations with recurrence and death. Although soluble CD137 and CD80 exhibited minimal variation and were therefore excluded from further analyses, both soluble CD28 (sCD28) and CTLA-4, which play distinct roles in T-cell activation, were associated with poor clinical outcomes in patients with ccRCC. Specifically, CTLA-4 was associated with an increased risk of recurrence, whereas sCD28 was associated with an increased risk of death. Furthermore, sCD28 was negatively correlated with the T-cell cytolytic score (rho = −0.33, *P* = 0.04) in primary ccRCC tumors. The authors suggested that interactions between sCD28, soluble CTLA-4 (sCTLA-4), and antitumor immunity may underlie their association with ccRCC outcomes, highlighting the potential prognostic value of these soluble immune checkpoint-related CD molecules [95].

CD163-positive M2 macrophages contribute to tumor-associated immune suppression by increasing PD-L1 and IL-10 expression. Soluble CD163 (sCD163), a marker of macrophage activation, has therefore been investigated as a potential biomarker in RCC. Indeed, in a study of patients with RCC, including a subgroup analysis of ccRCC, serum and urinary sCD163 levels were quantified by enzyme-linked immunosorbent assays, yielding similar findings in both cohorts. Serum sCD163 was detectable in all samples, and elevated serum concentrations were significantly associated with metastatic disease, suggesting that sCD163 may reflect macrophage-mediated immune modulation and tumor progression in ccRCC [86]. More recently, Lauridsen et al. first investigated soluble CD206 (sCD206), a marker of M2-like tumor-associated macrophage activation, as a potential biomarker in mRCC using the same assay previously employed for sCD163. Serum sCD206 levels were elevated in patients with intermediate-risk disease and correlated with other macrophage-related inflammatory markers, including sCD163 and C-reactive protein. Higher baseline sCD206 levels were associated with reduced OS (HR = 1.37, 95% CI: 1.12–1.67, *p* = 0.002), suggesting its prognostic potential. Moreover, the inclusion of sCD206 in established clinical risk models improved patient stratification, indicating its potential utility as an adjunct biomarker for monitoring and clinical management of patients with mRCC [108]. Nevertheless, larger prospective studies are needed to validate these findings and determine whether sCD206 can be implemented as a routine clinical biomarker.

### 3.2. Other Circulating and Urinary Soluble Protein Biomarkers 

Apart from immune-related biomarkers, several circulating and urinary proteins have been proposed as potential biomarkers in ccRCC, reflecting tumor progression, inflammation, angiogenesis, and host immune response. Proteomic studies and clinical cohorts have identified multiple circulating candidates with prognostic or predictive potential, demonstrating promising associations with disease outcomes in ccRCC, although their clinical utility requires additional validation [61,109,110].

Current evidence indicates that high tumor expression of mucin 1 (MUC1) in ccRCC is associated with an immunosuppressive TME characterized by reduced immune cell infiltration, increased M2 tumor-associated macrophage polarization, and lower PD-L1 expression. Additionally, in a cohort of 428 patients who underwent radical or partial nephrectomy for ccRCC, elevated circulating plasma levels of cancer antigen 15.3 (CA15.3), the soluble form of MUC1, were associated with nodal and visceral metastases, advanced tumor stage, and higher nuclear grade, serving as significant predictors of mortality risk. Serum CA15.3 levels were higher in patients with ccRCC compared with healthy participants and were associated with poorer CSS (HR 1.97, CI 1.08–2.94, *p* = 0.01). Moreover, CA15.3 remained an independent prognostic biomarker of PFS, highlighting its potential value for identifying patients with poor prognosis [111].

A proteomic study of circulating proteins in ccRCC identified 30 differentially abundant serum proteins associated with early-stage disease using paired immunodepletion and isobaric tags for relative and absolute quantification (iTRAQ)-based proteomic analysis. Heat shock cognate 71 kDa protein/heat shock protein family A (Hsp70) member 8 (HSC71/HSPA8) exhibited the greatest difference in abundance, while subsequent validation demonstrated significantly higher serum levels in patients with ccRCC than in healthy controls and patients with other urological diseases, suggesting that HSC71 may serve as a potential serum biomarker for ccRCC detection [112].

Similarly, another study identified 74 differentially abundant serum proteins between patients with early-stage ccRCC and healthy controls, of which 27 also differed significantly from patients with other urological malignancies and benign urological diseases. In addition, elevated urinary levels of soluble serpin family A member 1 (SERPINA1) and apolipoprotein H (APOH) were detected in patients with von Hippel–Lindau (VHL)-associated ccRCC [113]. Interestingly, integration of serum proteomic data with The Cancer Genome Atlas (TCGA) tissue gene expression profiles identified 11 overlapping proteins, including complement C1q B chain (C1QB), C1QC, annexin A1 (ANXA1), lysozyme (LYZ), S100 calcium binding protein A9 (S100A9), and serpin family A member 4 (SERPINA4). These proteins were associated with tumor stage and grade, underscoring their potential relevance as biomarkers for ccRCC progression and classification [114].

In the study by Wang et al. described above, the authors also evaluated circulating levels of the plasma immune checkpoint proteins soluble LAG-3 (sLAG-3), soluble TIM-3 (sTIM-3), and soluble B- and T-lymphocyte attenuator (sBTLA) in patients with ccRCC. They found that elevated circulating sLAG-3 levels were associated with an increased risk of advanced disease, whereas increased sTIM-3 and sBTLA levels correlated with a higher risk of death, suggesting their potential predictive value for treatment outcomes [95]. Likewise, heat shock protein 27 (Hsp27) was elevated in both urine and serum, with elevated serum Hsp27 levels being associated with high-grade tumors in patients with ccRCC, supporting its potential utility as a prognostic biomarker [115].

Given the central role of *VHL* mutations in ccRCC, researchers have also investigated several soluble proteins downstream of the hypoxia signaling pathway as potential biomarkers. Hypoxia-inducible protein 2 (HIG2) was reported to be approximately three-fold higher in the plasma of patients with RCC, with concentrations declining significantly after nephrectomy [116]. Similarly, circulating CAIX concentrations and enzymatic activity were increased in patients with ccRCC [117]. Elevated plasma levels of insulin-like growth factor II mRNA-binding protein 3 (IMP3) have also been reported and were associated with the development of distant metastases [118]. 

Several urinary protein biomarkers have also been identified for ccRCC prognosis and diagnosis. Increased urinary levels of haptoglobin (HP), fibronectin 1 (FN1), alpha-1,3-mannosyl-glycoprotein 4-beta-N-acetylglucosaminyltransferase A (MGAT4A), and apolipo-protein A1 (APOA1), together with decreased levels of kininogen 1 (KNG1), uromodulin (UMOD), apolipoprotein D (APOD), CD59, and heparan sulfate proteoglycan 2 (HSPG2), have been associated with poor prognosis in ccRCC [119]. Other urinary proteins, including UMOD, RAS-like proto-oncogene A (RALA), and carnosine dipeptidase 1 (CNDP1), were associated with venous invasion, whereas HP, lumican (LUM), cornulin (CRNN), and annexin A2 (ANXA2) increased progressively with tumor grade [120]. A glycoproteomic analysis further demonstrated increased urinary abundance of CD97, cochlin (COCH), and phosphoinositide-3-kinase interacting protein 1 (PIK3IP1) with advancing tumor stage, whereas apolipoprotein B (APOB), FN1, ceruloplasmin (CERU), complement factor H (CFH), HP, and phospholipid transfer protein (PLTP) were decreased compared with healthy controls [121]. In addition, urinary glutaredoxin (GLRX), cystatin C (CST3), solute carrier family 9 member A3 regulator 1 (SLC9A3R1), heat shock protein family E (Hsp10) member 1 (HSPE1), FKBP prolyl isomerase 1A (FKBP1A), eukaryotic translation elongation factor 1 gamma (EEF1G), chromosome 12 open reading frame 49 (C12orf49), EH domain containing 4 (EHD4), EPS8 like 2 (EPS8L2), charged multivesicular body protein 2A (CHMP2A), programmed cell death 6 interacting protein (PDCD6IP), carnosine dipeptidase 2 (CNDP2), CEA cell adhesion molecule 1 (CEACAM1), and chaperonin containing TCP1 subunit 6A (CCT6A) have been proposed as biomarkers for distinguishing ccRCC from oncocytoma, detecting early-stage disease, and predicting disease progression [122]. Table 2 summarizes all circulating and urinary protein biomarkers in ccRCC.

## 4. Immunotherapy and Predictive Markers in ccRCC 

**Figure 2:**
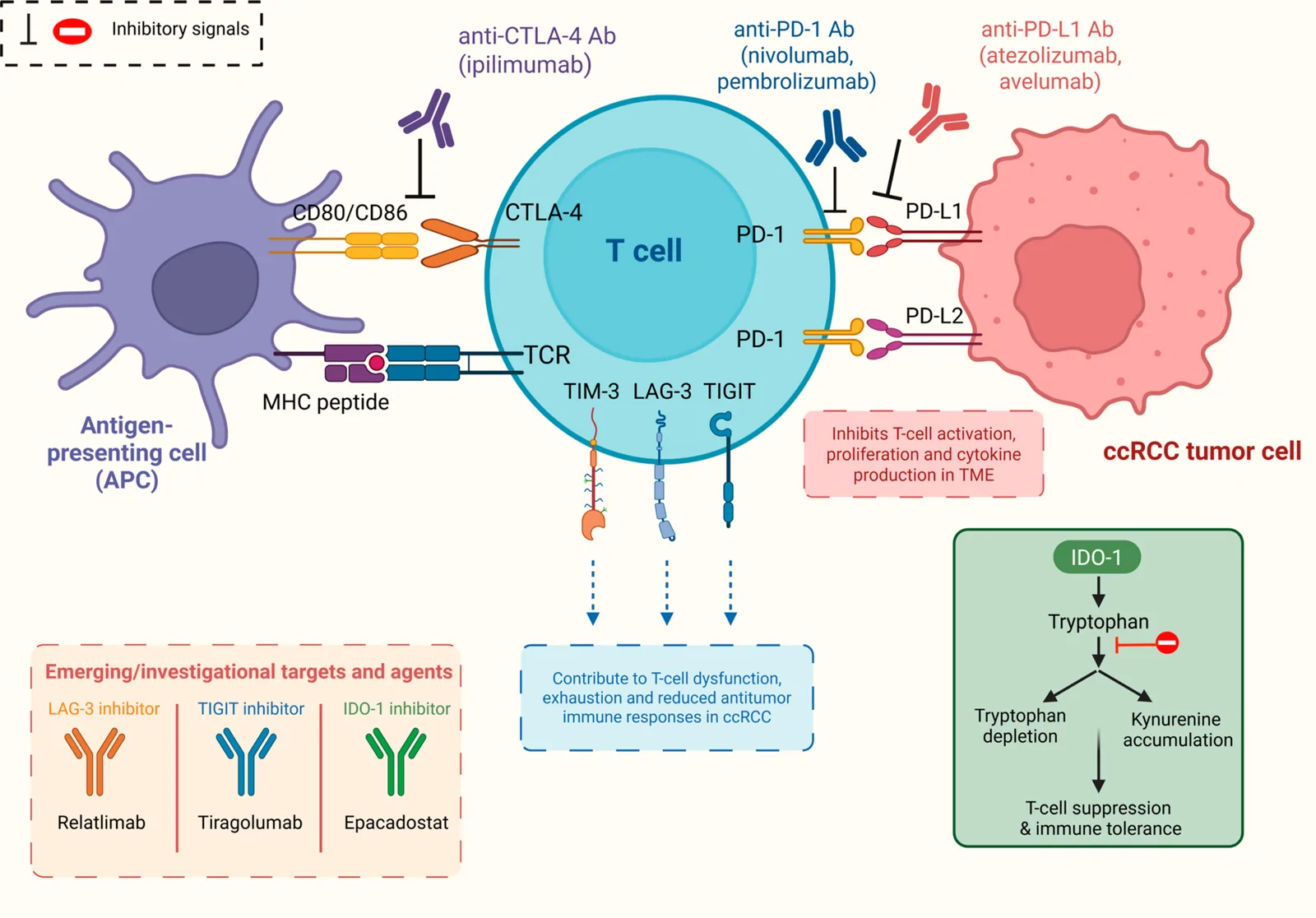
Key immune checkpoint molecules and therapeutic targets in clear cell renal cell carcinoma (ccRCC). Key inhibitory immune checkpoint pathways involving CTLA-4, PD-1/PD-L1/PD-L2, TIM-3, LAG-3 and TIGIT are presented, along with IDO-1. Established immune checkpoint inhibitors targeting CTLA-4, PD-1 and PD-L1 are demonstrated together with emerging or investigational regimens targeting LAG-3, TIGIT and IDO-1. Activation of these inhibitory pathways results in reduced T-cell activation, T-cell dysfunction, and suppression of antitumor immune responses within the tumor microenvironment. Created in BioRender. Anatoliotaki, C. (2026) https://BioRender.com/w6rogq3. Abbreviations: APC, antigen-presenting cell; ccRCC, clear cell renal cell carcinoma; CTLA-4, cytotoxic T-lymphocyte-associated protein 4; IDO-1, indoleamine 2,3-dioxygenase 1; LAG-3, lymphocyte-activation gene 3; MHC, major histocompatibility complex; PD-1, programmed cell death protein 1; PD-L1, programmed death-ligand 1; PD-L2, programmed death-ligand 2; TCR, T-cell receptor; TIGIT, T-cell immunoreceptor with immunoglobulin and ITIM domains; TIM-3, T-cell immunoglobulin and mucin-domain containing-3.

Over the past decade, scientific interest has focused on developing and investigating new therapeutic strategies that target the immune system [123]. These approaches have transformed the therapeutic landscape of cancer treatment, particularly in RCC, melanoma, and non-small-cell lung cancer (NSCLC) [31]. Historically, cytokine-based therapies, including interleukin-2 (IL-2) and recombinant interferon-α (IFN-α), were among the first immunotherapies approved for ccRCC. Although these agents produced durable responses in a small subset of patients, their clinical application was limited due to low efficacy and high toxicity rates [124]. Consequently, management of advanced RCC evolved from non-specific immunotherapeutic approaches tο targeted therapies directed against angiogenic pathways, including VEGF and its receptor VEGFR [125]. More recently, ICIs have emerged as a major therapeutic option for treating patients with ccRCC. These agents primarily target checkpoint proteins such as PD-1, PD-L1, and CTLA-4 [33]. ICIs, including nivolumab, pembrolizumab, and ipilimumab, block inhibitory signaling pathways that negatively regulate T-cell activity, restoring antitumor immune responses and enhancing the immune system’s ability to eliminate tumor cells [41]. The increasing therapeutic use of ICIs has led to significant clinical benefits in both primary and metastatic ccRCC, while combinatorial approaches demonstrate a beneficial clinical outcome across diverse patient cohorts [33,124]. To better understand their therapeutic role in ccRCC, the following sections provide a detailed examination of the major inhibitors. Figure 2 summarizes the main immune checkpoint pathways involved in ccRCC, along with established and emerging therapeutic targets.

### 4.1. PD-1 Inhibitors

Inhibitors of PD-1 refer to monoclonal antibodies that block PD-1 receptor expressed on T cells [33]. Under physiological conditions, interaction between PD-1 and its ligand, PD-L1, leads to suppression of T-cell activation via signaling pathways, including PI3K-AKT-mTOR (mechanistic target of rapamycin) [44]. Such inhibitors established themselves as important therapeutic agents since blocking of the PD-1/PD-L1 interaction reactivates T-cell proliferation and promotes antitumor immunity [41]. Nivolumab is a human immunoglobulin G4 (IgG4) monoclonal antibody [124]. Notably, evidence from the phase III CheckMate 025 trial demonstrated significant antitumor activity and improved OS compared with everolimus, an mTOR inhibitor (25.0 vs. 19.6 months), while also achieving a higher objective response rate (ORR) (25% vs. 5%) in patients with advanced ccRCC [126]. On 2015, the U.S. Food and Drug Administration (FDA) approved nivolumab as the first immune checkpoint inhibitor for the treatment of patients with advanced RCC [127]. 

Pembrolizumab is the first humanized monoclonal IgG4 antibody targeting PD-1 [124] and was investigated as a monotherapy in the phase II KEYNOTE-427 trial (NCT02853344) in patients with advanced ccRCC, with promising clinical outcomes. This trial demonstrated favorable 12- and 24-month OS rates for most patients, along with a beneficial PFS outcome and an ORR of 36.4% [44]. Although monotherapy with pembrolizumab was associated with a positive clinical performance in this trial, subsequent clinical development in RCC has primarily focused on combination-based regimens, which have demonstrated clinical benefit and received FDA approval for treating advanced RCC [25,128].

### 4.2. PD-L1 Inhibitors

Similarly, PD-L1 inhibitors also act through disruption of the PD-L1 and PD-1 interaction, thereby restoring T-cell cytotoxic activity and enhancing tumor-induced immunity [41,123]. Among these agents, atezolizumab is a monoclonal antibody examined in the phase II IMmotion150 trial (NCT01984242) as a first-line treatment for patients with advanced mRCC with clear cell histology and/or a component of sarcomatoid histology [44]. The trial demonstrated clinical activity, with an ORR of 25% and a median PFS of 6.1 months, although these outcomes were not superior to those observed with the RTK inhibitor sunitinib, which achieved an ORR of 29% and a median PFS of 8.4 months [33]. Avelumab is another monoclonal antibody against PD-L1 that has been tested in combination with axitinib, showing favorable clinical effects [24,41]. The strong efficacy of PD-L1 blockade has supported further investigation of combination therapies in ccRCC [123].

### 4.3. CTLA-4 Inhibitors

Ipilimumab is a human immunoglobulin G1 (IgG1) monoclonal antibody, targeting CTLA-4 and antagonizing its interaction with co-stimulatory ligands CD80/CD86, thereby promoting T-cell activation [41,124]. The therapeutic potential of ipilimumab was investigated in the phase II clinical trial NCT00057889, where ipilimumab showed overall response rates of 5% and 12.5% depending on the dosing regimen. However, it should be noted that treatment was also associated with considerable immune-related toxicity (43% of patients receiving the high-dose regimen) [129]. Although ipilimumab monotherapy has limited efficacy in patients with advanced RCC and metastatic ccRCC, clinical studies have shown that its combination with nivolumab improved OS. Unfortunately, these benefits are accompanied by increased immune-related adverse events [37], raising questions of whether all patients truly benefit from combination therapy given the associated toxicities. 

### 4.4. LAG-3 Inhibitors

Relatlimab, a human IgG4 monoclonal antibody that targets LAG-3, was first approved by FDA for the treatment of melanoma [124,130] and is currently being evaluated in an ongoing phase II clinical trial (NCT06708949) as part of a first-line treatment combination with nivolumab and ipilimumab for RCC with a clear cell component (ClinicalTrials.gov NCT06708949). Although the therapeutic potential of relatlimab in advanced RCC awaits clinical evaluation, this strategy is based on the observation that multiple tumors, including ccRCC, that simultaneously exploit the inhibitory function of LAG-3 and PD-1 to suppress antitumor immunity [50]. 

### 4.5. TIGIT Inhibitors

Similar to other immune checkpoint molecules, TIGIT is expressed on multiple immune cell types, including activated CD8+ and CD4+ T cells. Among its ligands, CD155 binds preferentially to TIGIT, resulting in reduced cytokine production and decreased T-cell activation [44,73]. Tiragolumab is a monoclonal IgG4 antibody that blocks TIGIT and is in phase II clinical trials (NCT05805501) for metastatic or advanced ccRCC in combination with axitinib and tobemstomig (ClinicalTrials.gov NCT05805501). Tiragolumab represents a promising therapeutic agent for ccRCC, as simultaneous blockade of TIGIT and PD-1 has been associated with enhanced T-cell proliferation and cytokine production [73].

### 4.6. Emerging Therapeutic Targets

Alongside the established ICIs, several other immune-related molecules have been investigated as attractive therapeutic targets for treating RCC [131]. Among them, IDO-1, an enzyme crucial to tryptophan metabolism, promotes an immunosuppressive tumor microenvironment [132]. Epacadostat, a selective inhibitor of IDO-1, was evaluated in combination with pembrolizumab in patients with metastatic or advanced ccRCC in the phase III KEYNOTE-679 clinical trial (NCT03260894) [133]. However, clinical results showed limited additional benefit compared with existing therapeutic strategies, with an ORR of 31.3% versus 29.2% for sunitinib, although additional research in carefully stratified patient cohorts is required [133]. Another immune checkpoint regulator, V-domain immunoglobulin suppressor of T-cell activation (VISTA), has shown antitumor activity in preclinical RCC models, where VISTA-targeting antibodies resulted in reduced tumor growth, supporting its potential as a promising therapeutic target in ccRCC [44]. Notably, TIM-3 has attracted growing interest because of its role in T-cell exhaustion and immune resistance. However, evidence regarding sabatolimab (TIM-3 blockade) in RCC and ccRCC remains scarce, and additional studies are required to determine its therapeutic potential [134]. Beyond immune checkpoint molecules, ectonucleotide pyrophosphatase/phosphodiesterase 3 (ENPP3) has emerged as a future therapeutic target in ccRCC due to its high expression in RCC and limited expression in normal tissues. XmAb819 is an ENPP3xCD3 bispecific T-cell engager currently under investigation in the phase I clinical trial NCT05433142 in patients with advanced ccRCC. Early findings have shown an ORR of 25% and a disease control rate of 70% in patients previously treated with multiple lines of therapy, supporting the potential to enhance a restored antitumor immune activity [135,136]. Another novel target is CD70, which is frequently expressed in RCC, particularly ccRCC, while showing low expression in normal tissues. ALLO-316, an allogeneic CD70-targeted chimeric antigen receptor (CAR) T-cell therapy, was studied in the phase I TRAVERSE trial (NCT04696731) in patients with advanced ccRCC, with an ORR of 25.0% observed in the phase Ib, increasing to 31.3% among patients with high CD70 expression [137]. Moreover, human endogenous retrovirus type E (HERV-E) is a suitable target for T-cell-based therapies because of its high expression in ccRCC. In the phase I clinical trial NCT03354390 in patients with metastatic ccRCC, HERV-E T cells were investigated, with partial responses reported in 7% of participants and stable disease lasting at least 8 weeks in 29% of the cohort [138]. Table 3 summarizes the main immune checkpoint-targeted therapies and emerging therapeutic targets discussed above.

Taken together, ICIs have revolutionized the therapeutic landscape of RCC, demonstrating significant clinical benefits across a variety of patient populations and disease stages [31]. However, although these agents have produced encouraging results, some patients do not respond and develop resistance. Furthermore, the emergence of side effects, such as immune-related adverse events, and the biological heterogeneity of renal tumors, remain major challenges in clinical practice. Consequently, these limitations have driven the development of combination-based therapeutic strategies to improve clinical responses [139]. 

## 5. Combined Strategies and New Therapeutic Approaches 

To improve clinical outcomes and overcome treatment resistance and limitations, recent studies in ccRCC treatment have focused on combinatorial strategies involving either multiple immune checkpoint blockers or ICIs combined with non-immunotherapeutic agents [124]. These approaches may provide additional therapeutic benefits compared with monotherapy, particularly in patients with advanced ccRCC or poor prognostic features [125]. Current ccRCC management includes combination-based first-line strategies [39], while ongoing clinical trials continue to explore [39] their therapeutic potential [44]. 

### 5.1. Combination of ICIs

**Figure 3:**
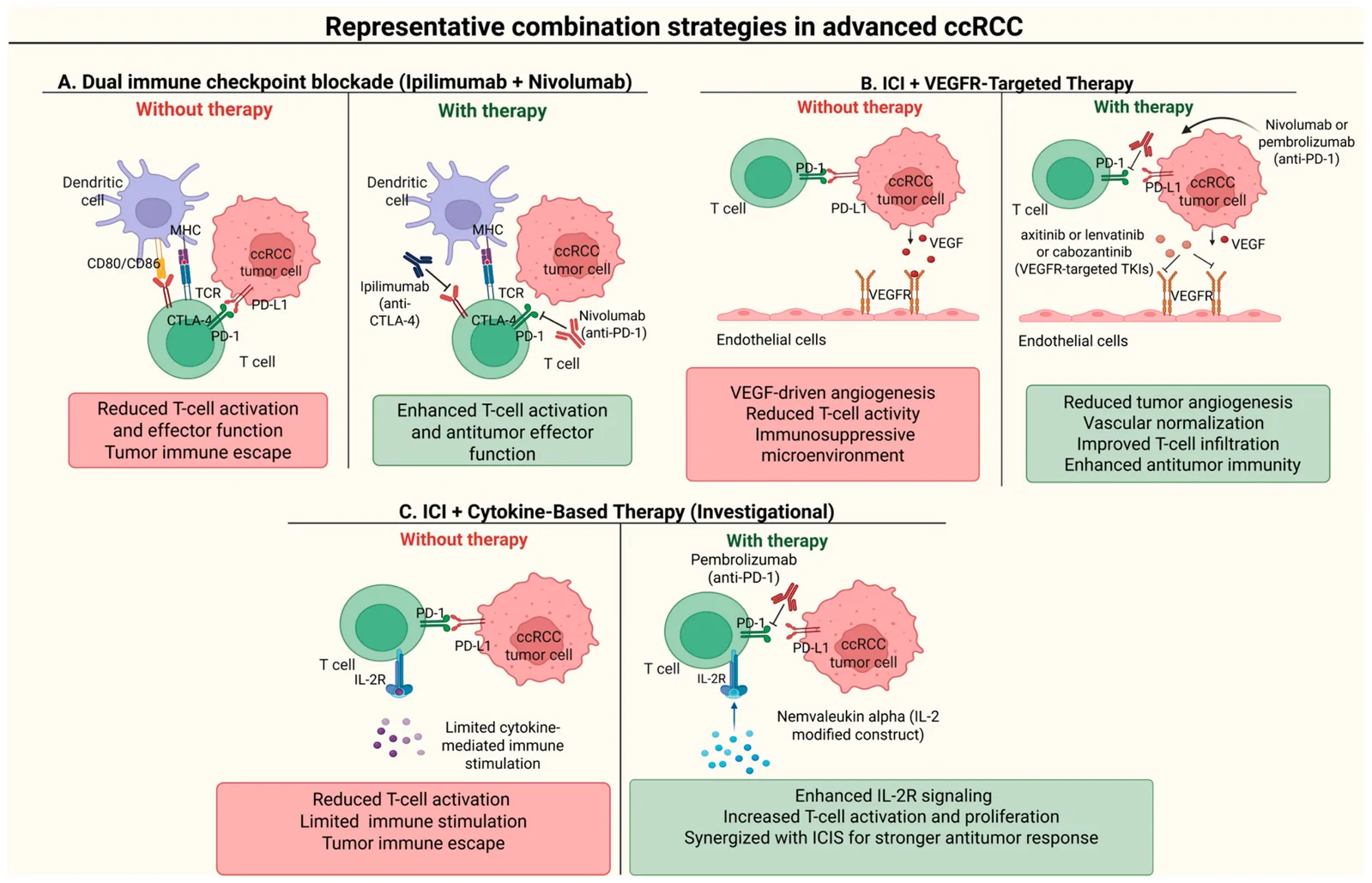
Representative combination strategies in advanced clear cell renal carcinoma (ccRCC). (**A**) Dual immune checkpoint blockade with ipilimumab plus nivolumab, targeting the CTLA-4 and PD-1 pathways, promotes T-cell activation and antitumor effector function. (**B**) ICI combined with VEGFR-targeted therapy limits VEGF-driven angiogenesis, enhances vascular normalization, and improves T-cell infiltration. (**C**) Investigational cytokine-based therapy with pembrolizumab plus modified IL-2, nemvaleukin alpha, promotes IL-2R signaling and antitumor immune responses. Created in BioRender. Anatoliotaki, C. (2026) https://BioRender.com/vam0kwc. Abbreviations: ccRCC, clear cell renal cell carcinoma; CTLA-4, cytotoxic T-lymphocyte-associated protein 4; ICI, immune checkpoint inhibitor; IL-2R, interleukin-2 receptor; MHC, major histocompatibility complex; PD-1, programmed cell death protein 1; PD-L1, programmed death-ligand 1; TCR, T-cell receptor; VEGF, vascular endothelial growth factor; and VEGFR, vascular endothelial growth factor receptor.

In 2018, the FDA approved the combination therapy of nivolumab with ipilimumab as an initial treatment for patients with advanced ccRCC with intermediate- or poor-risk disease [37]. The clinical benefit was based on the complementary mechanisms of PD-1 and CTLA-4 blockade. While PD-1 inhibition reverses T-cell exhaustion, CTLA-4 blockade enhances antigen-specific T-cell activation [124]. The CheckMate 214 trial investigated this regimen and showed a significant OS benefit: 18-month OS was 75% versus 60% with sunitinib monotherapy, with a higher ORR (42% vs. 27%) [44]. Although multiple participants achieved greater tumor reduction and a better quality of life with this strategy, side effects including diarrhea and hypertension were also recorded [125]. In addition to the nivolumab–ipilimumab administration, ongoing clinical trials are also evaluating emerging dual-checkpoint strategies [31]. Notably, a phase II trial (NCT06708949) is studying the addition of relatlimab (LAG-3 inhibitor) to nivolumab–ipilimumab for treating advanced ccRCC [140]. Figure 3 summarizes representative combination strategies in advanced ccRCC, including dual immune checkpoint blockade, ICI-VEGFR-targeted therapy, and investigational ICI-cytokine-based approaches.

### 5.2. Combination of ICIs and Anti-VEGF Therapies 

Currently, multiple combinations of ICIs with anti-VEGF therapies have transformed the treatment paradigm for RCC, with evidence derived from several pivotal clinical trials, including IMmotion151, JAVELIN Renal 101, CLEAR/KEYNOTE-581, CheckMate 9ER and KEYNOTE-426 [44]. Through promotion of vascular normalization and reduction tumor immunosuppression, VEGF inhibitors can enhance T-cell infiltration and improve treatment efficacy [124]. 

In the phase III CheckMate 9ER trial (NCT03141177), a combination of nivolumab with cabozantinib demonstrated improved PFS (16.6 vs. 8.3 months), OS, and ORR (55.7% vs. 27.1%) compared with sunitinib in untreated patients with advanced ccRCC. Based on these results, this strategy received FDA approval in 2021 as a first-line treatment for this patient cohort [44]. The 5.6-year follow-up analysis confirmed the durability of these clinical benefits, with nivolumab plus cabozantinib maintaining survival and response advantages over sunitinib [141]. Another important VEGF-ICI combination is pembrolizumab plus axitinib [33]. The phase III KEYNOTE-426 trial (NCT02853331) highlighted this strategy for the improved survival outcome compared to sunitinib, including a higher ORR (59.3% vs. 35.7%), prolonged median PFS (15.1 vs. 11.1 months) and improved 12-month OS (89.9% vs. 78.3%), establishing it as a first-line therapeutic option for advanced ccRCC, leading to its approval by FDA in 2019 [128]. However, it should be noted that treatment was associated with several adverse effects, including hepatotoxicity, nausea, and hypertension [142]. Similarly, axitinib combined with avelumab has been evaluated as a treatment strategy in patients with advanced RCC with a clear-cell component [44]. In the phase III JAVELIN RENAL 101 (NCT02684006) clinical trial, this combination was compared against sunitinib therapy, demonstrating a significant improvement in PFS (13.0 vs. 8.4 months), and a higher ORR (55.2% vs. 25.5%), while OS data were not ready at the time of primary analysis [24]. Nevertheless, FDA approved this combination in 2019 as a first-line treatment for advanced RCC [143]. Although an updated analysis confirmed the durability of the PFS benefit with almost doubled ORR rates, no significant OS advantage has been reported compared to the previously mentioned strategies [144]. 

Additional VEGF–ICI combinations have also demonstrated promising efficacy in advanced RCC. In the phase III CLEAR/KEYNOTE-581 trial (NCT02811861), the combination of pembrolizumab with lenvatinib was assessed in patients with advanced RCC with a clear-cell component and was associated with prolonged median PFS (23.9 vs. 9.2 months) and improved OS (hazard ratio 0.66) compared with sunitinib [44]. However, grade 3 adverse effects were more frequent (82.4% vs. 71.8%), including hypertension and diarrhea [145]. Based on these results, pembrolizumab plus lenvatinib received FDA approval in 2021 [25]. Furthermore, the IMmotion151 trial (NCT02420821), which included untreated patients with advanced or metastatic RCC with clear-cell histology or sarcomatoid component, showed that atezolizumab combined with bevacizumab improved PFS compared with sunitinib, particularly in selected patient cohorts (11.2 vs. 7.7 months along with a hazard’s ratio of 0.75) [44]. The combination was also associated with a lower incidence of grade 3–4 adverse events (40% vs. 54%), supporting a more favorable safety profile [146]. However, the final OS analysis showed no significant benefit (36.1 vs. 35.5 months) and the findings of the IMmotion151 trial did not lead to FDA approval for RCC treatment [147]. Beyond these established regimens, several emerging strategies, including toripalimab plus axitinib, sitravatinib plus nivolumab [124], and triplet combinations with cabozantinib, nivolumab, and ipilimumab, have shown promising therapeutic effects [33]. However, not all VEGF–ICI combinations have demonstrated consistent clinical benefits, especially in patients previously exposed to immune checkpoint blockade [33]. Consequently, phase III clinical trials have shown that VEGF-ICI combination-based therapies have transformed the therapeutic management of advanced ccRCC and currently represent preferred first-line treatment (summarized in Table 4). Although these regimens have increased response rates and PFS compared with monotherapies, ongoing clinical trials and the development of predictive biomarkers remain essential for patient selection [131].

### 5.3. ICIs Combined with Cytokine Therapies

As previously mentioned, application of cytokine therapies, particularly high-dose IL-2, has decreased due to severe toxicity [31]. More recently, cytokine-based regimens have also been explored in combination with immune checkpoint blockers [44], with a focus on developing modified cytokines such as nemvaleukin alfa, a novel IL-2 construct [148], and pegilodecakin, a pegylated recombinant IL-10 [149]. Both agents have been investigated with pembrolizumab in the phase I/II ARTISTRY-1 (NCT02799095) and phase I/Ib IVY (NCT02009449) clinical trials, respectively, in RCC cohorts with a high rate of clear-cell histology [148,149]. ARTISTRY-1 demonstrated promising antitumor activity and an acceptable safety profile, supporting the need for additional clinical evaluation of nemvaleukin alfa in combination with ICIs [148]. Likewise, results from the phase I/Ib IVY trial showed that pegilodecakin combined with either pembrolizumab or nivolumab was associated with encouraging response rates (ORR of 43% and median PFS of 13.9 months), enhancing the antitumor activity of PD-1 inhibitors [149]. Of note, most participants (68%) experienced grade 3–4 adverse events, including thrombocytopenia (21%) and hypertriglyceridemia (16%) [150]. 

Beyond nemvaleukin alfa and pegilodecakin, another modified cytokine that has been investigated in combination with nivolumab is bempegaldesleukin (NKTR-214). Bempegaldesleukin is a pegylated IL-2 agonist designed to preferentially activate CD8^+^ T cells despite the presence of immunosuppressive regulatory T cells in the TME. Results from the phase I PIVOT-02 (NCT02983045) clinical trial, with an ORR of 71.4% and median PFS of 14.2 months, revealed promising efficacy of this regimen [151]. However, the phase III PIVOT-09 trial (NCT03729245) compared bempegaldesleukin plus nivolumab with tyrosine kinase inhibitor (TKI)-based therapies and did not confirm the potential of this strategy in patients with untreated advanced RCC, with an ORR of 23.0% compared with 30.6% with TKI-based therapy [152]. Despite encouraging results from early-phase studies, cytokine-based combination agents have not yet been translated into FDA-approved therapies for RCC [148,152]. 

### 5.4. Alternative Therapeutic Options

Beyond the currently established treatment strategies, multiple emerging therapeutic options are under investigation in RCC management. One notable development involves agents targeting HIF-2a, a key regulator of the VHL-HIF signaling pathway that contributes to ccRCC pathogenesis. One representative agent in this class is belzutifan, which has shown favorable antitumor activity and is currently being explored in several clinical trials, including LITESPARK-011 (NCT04586231) and LITESPARK-012 (NCT04736706), to improve clinical outcomes in advanced ccRCC [153]. An additional area of interest involves metabolism-targeting inhibitors. The phase II ENTRATA trial (NCT03163667) showed encouraging clinical activity for telaglenastat, a glutaminase inhibitor, in combination with everolimus, an mTOR inhibitor, in patients with advanced RCC with clear-cell component (PFS of 3.8 vs. 1.9 months). However, these findings were not replicated in the phase II of the CANTATA trial (NCT03428217), which examined telaglenastat in combination with cabozantinib and failed to demonstrate an improvement in PFS (9.2 vs. 9.3 months) [153,154]. An overview of emerging combination strategies currently under clinical investigation for advanced RCC is provided in Table 5. 

Collectively, these lines of evidence underscore the opportunities and limitations of exploring new therapeutic agents in ccRCC. Future directions may focus on treatment strategies with biomarker-guided patient selection to achieve a more personalized approach to therapy and maximize long-term clinical benefit in patients with ccRCC [131].

## 6. Conclusions and Future Perspectives 

Immune-related proteomic profiling in clear cell renal cell carcinoma (ccRCC) represents a promising approach to identifying biomarkers that may improve disease characterization, prognostic assessment, and treatment selection. Proteins detected in tumor tissue or circulating biological fluids, including PD-L1, PD-1, CTLA-4, TIM-3, KIM-1, VEGF, IL-6, and IL-8, have shown associations with clinical outcomes or treatment-related variables in selected cohorts. However, these findings should be interpreted cautiously, as the clinical validity and utility of most candidate biomarkers remain insufficiently established. To date, no single immune-related protein has demonstrated sufficiently consistent and independently validated predictive performance to support its use as a standalone biomarker for selecting patients with ccRCC for immunotherapy.

Several important limitations hinder the translation of these findings into clinical practice. First, ccRCC is characterized by substantial interpatient and intratumoral heterogeneity, including variation in tumor molecular features, immune-cell composition, immune checkpoint expression, and the tumor microenvironment. Consequently, a biomarker measured in a single tissue specimen may not adequately represent the biological diversity of the entire tumor or metastatic sites. Spatial and temporal changes, including those occurring during treatment, may further affect biomarker expression and interpretation. These factors complicate comparisons between cohorts and may limit the reproducibility of associations observed in individual studies.

Second, the available evidence for many candidate biomarkers is derived from relatively small cohorts, retrospective analyses, or exploratory studies with heterogeneous patient populations, treatment regimens, sampling procedures, and clinical endpoints. Such designs are vulnerable to selection bias, confounding, and overfitting, particularly when multiple biomarkers or cutoffs are evaluated. Moreover, statistically significant associations with tumor stage, recurrence, or survival do not necessarily establish diagnostic accuracy or demonstrate that a biomarker predicts differential benefit from a particular treatment. Predictive claims require appropriately designed studies that evaluate treatment-specific effects, ideally including biomarker-by-treatment interactions. Independent validation in adequately powered, prospective, and clinically representative cohorts remains essential before proposed biomarkers or multianalyte signatures can be considered clinically actionable.

A further challenge is the lack of standardization across proteomic assays and study protocols. Differences in specimen collection and processing, storage conditions, analytical platforms, antibody specificity, normalization procedures, and data analysis may contribute to inconsistent results. Immunohistochemical assessment of tissue biomarkers may be affected by antibody selection, scoring systems, intratumoral sampling, and the thresholds used to define positivity, whereas circulating protein measurements may vary according to assay sensitivity, calibration, and preanalytical handling. Accordingly, cutoffs derived from individual cohorts should not be assumed to be transferable across laboratories or patient populations without appropriate analytical and clinical validation. Harmonized protocols, transparent reporting, and reproducibility studies are needed to establish reliable reference ranges and clinically meaningful thresholds.

The interpretation of circulating biomarkers warrants particular caution because many are not specific to ccRCC or to tumor-associated immune activity. KIM-1 concentrations may be influenced by renal injury and kidney function, while VEGF can reflect angiogenic processes beyond the tumor itself. IL-6 and IL-8 are inflammatory mediators that may increase in response to systemic inflammation, infection, comorbid conditions, or other malignancies. In addition, hemolysis and other preanalytical factors may alter measured concentrations of certain circulating proteins, depending on the analyte and assay. Renal function, inflammatory status, sample quality, and relevant clinical covariates should therefore be considered when interpreting circulating biomarker measurements. Without appropriate controls and adjustment for these factors, an observed association with disease status or outcome may not be attributable specifically to ccRCC biology. Serial measurements may ultimately prove useful for monitoring individual patients, but their interpretation will require standardized sampling and evidence that observed changes reflect clinically meaningful tumor dynamics rather than fluctuations in renal function, inflammation, or sample handling.

Future research should therefore prioritize rigorous analytical validation, prospective multicenter studies, and independent replication using harmonized protocols and prespecified endpoints and cutoffs. Combining tissue-based and circulating proteomic markers with clinicopathological variables and established molecular features may help capture the complexity of ccRCC more effectively than any single protein. Integration with spatial and single-cell profiling, transcriptomics, genomics, and metabolomics may further clarify the relationships between tumor heterogeneity, immune contexture, and treatment response. Artificial intelligence and machine-learning approaches may also support the development of multivariable models, provided that these models are trained on sufficiently large datasets and evaluated in independent cohorts to limit overfitting and ensure generalizability [155]. Liquid biopsy-based proteomics offers a potentially minimally invasive means of longitudinal assessment, but its ability to guide treatment decisions or improve patient outcomes remains to be demonstrated in prospective clinical studies [109,156]. 

Overall, immune-related proteomic biomarkers offer important research opportunities in ccRCC, but their clinical promise should not be equated with established clinical utility. Progress toward personalized management will depend not only on discovering new candidate proteins, but also on demonstrating their analytical reliability, biological specificity, incremental value over existing clinical and pathological factors, and reproducible performance in independent patient populations. A more cautious, validation-driven approach will be essential to determine which biomarkers can ultimately support diagnosis, prognostic assessment, treatment selection, or disease monitoring in routine clinical practice. 

## Figures and Tables

**Table 1:** Immune checkpoint-related and immune-related protein biomarkers with clinical significance in clear cell renal cell carcinoma (ccRCC).

Biomarker	Category	Molecular Function	Clinicopathological Association	Line of Evidence	Utilization	Method/Cutoff	References
PD-L1	Immune checkpoint-related	Ligand of PD-1	Higher tumor grade, advanced TNM stage, metastasis	Protein (IHC)	Prognostic	PD-L1 positivity: 20.9%; OS: HR 2.0, 95% CI 1.2–3.3, *p* = 0.005	[30,36]
PD-1	Immune checkpoint-related	Cell surface immune checkpoint receptor	Advanced stage, sarcomatoid differentiation	Protein (IHC)	Prognostic	DMR: HR 6.146, 95% CI, 1.249–30.238, *p* = 0.026; shorter OS: HR 3.195, 95% CI 1.046–9.761, *p* = 0.042	[38,40]
PD-L2	Immune checkpoint-related	Ligand of PD-1	Lymph node metastasis	Protein (IHC)	Prognostic	DFS/PFS HR 1.45, 95% CI = 1.03–2.03, *p* = 0.033	[41,43]
CTLA-4	Immune checkpoint-related	Immune checkpoint protein involved in regulation of T-cell responses	Advanced stage, metastatic disease	mRNA (RNA-seq)	Prognostic	poor OS: HR 1.458, 95% CI 1.13–1.881, *p* = 0.004	[45,47]
TIM-3	Immune checkpoint-related	Associated with T-cell exhaustion and establishment of immunosuppressive TME	PD-1 co-expression, advanced TNM stage, aggressive tumor phenotype	Protein (IHC)	Prognostic	TIM-3 positivity: 69.2%, shorter PFS	[41,49]
LAG-3	Immune checkpoint-related	Cell surface immune checkpoint receptor	Higher TNM stage and higher Fuhrman nuclear grade	mRNA (RNA-seq)	Prognostic	OS: HR 1.236; PFS: HR 1.121; DSS: HR 1.269 (*p* < 0.05)	[31,51]
IDO-1	Immune checkpoint-related	Immunoregulatory enzyme involved in tumor immunosurveillance escape	Shorter OS in RCC	Protein	Potential prognostic	-	[41]
DNASE1L3	Immune-related	Tumor-suppressive protein	Higher expression is associated with lower tumor stage, tumor weight, clinical stage, histological grade	mRNA (RNA-seq)	Prognostic/potential diagnostic	Tumor vs. normal expression: 7.98 vs. 10.87, *p* < 0.001; tumor size: r = −0.32, *p* < 0.001	[52]
C1QC	Immune-related	Associated with immune-cell infiltration and modulation of TME	Advanced stage, lymph node metastasis	mRNA (RNA-seq)	Prognostic	OS: HR 1.67, 95% CI 1.23–2.27, *p* = 0.00097	[53]
MZT2A	Immune-related	Associated with immune-cell infiltration and modulation of TME	Advanced T-stage, M-stage, pathological grade	mRNA (transcriptomic analysis)	Prognostic/potential diagnostic	High MZT2A expression: shorter OS (*p* < 0.05)	[54]
Vimentin	Immune-related	Intermediate filament protein involved in EMT and cell migration	Vascular invasion, lymph node involvement, distant metastasis	Protein (IHC)	Prognostic	vascular invasion: OR 2.90, 95% CI 1.05–7.62, *p* = 0.031, distant metastatic spread: OR 3.10, 95% CI 1.01–12.54, *p* = 0.048, greater tumor size (*p* = 0.004)	[55]
RUNX1	Immune-related	Transcription factor involved in angiogenesis, immune regulation and lipid metabolism	Aggressive tumor behavior	Protein (IHC)	Prognostic	poor prognosis (HR 1.58, 95% CI 1.054–2.371, *p* = 0.027)	[57] (54)
CAIX	Immune-related	Hypoxia-related transmembrane enzyme involved in pH regulation	Lower tumor stage and improved survival outcomes	Protein (IHC)	Prognostic/Diagnostic	-	[58]
Ki-67	Immune-related	Marker of cellular proliferation with a crucial role in mitosis	Adverse pathological features, including sarcomatoid differentiation and a more aggressive tumor phenotype	Protein (IHC)	Prognostic	Ki-67 ≥ 30%: 21.3% of patients; worse RFS, CSS (*p* < 0.001)	[59]
E-cadherin, P-cadherin, N-cadherin	Immune-related	Cell-adhesion proteins involved in EMT	Lymph node metastasis, high tumor size, higher nuclear grade and advanced stage	Protein (IHC)	Prognostic	-	[60]

Abbreviations: C1QC, complement C1q C chain; CAIX, carbonic anhydrase IX; CI, confidence interval; CSS, cancer-specific survival; CTLA-4, cytotoxic T-lymphocyte-associated protein 4; DFS/PFS, disease-free survival/progression-free survival; DMR, distant metastatic relapse; DNASE1L3, deoxyribonuclease 1-like 3; DSS, disease-specific survival; EMT, epithelial–mesenchymal transition; HR, hazard ratio; IDO-1, indoleamine 2,3-dioxygenase 1; LAG-3, lymphocyte activation gene 3; MZT2A, mitotic spindle organizing protein 2A; OR, odds ratio; OS, overall survival; PD-1, programmed cell death protein 1; PD-L1, programmed death-ligand 1; PD-L2, programmed death-ligand 2; RCC, renal cell carcinoma; RFS, recurrence-free survival; RUNX1, runt-related transcription factor 1; TIM-3, T-cell immunoglobulin and mucin-domain containing-3; TME, tumor microenvironment; and TNM, tumor node metastasis.

**Table 2:** Circulating and urinary protein biomarkers in clear cell renal cell carcinoma (ccRCC).

Biomarker	Biological Material	Clinicopathological Association	Method/Cut-Off/Clinical Evidence	Utilization	Independent Validation	References
KIM-1	Plasma	Elevated in high-grade and metastatic ccRCC, associated with worse DFS/OS, shorter STFS	DFS: survival time ratio 0.56 (95% CI 0.42–0.73, *p* < 0.001); median DFS: 35.9 vs. 57.2 months (HR 1.75, 95% CI 1.40–2.17); atezolizumab benefit: HR 0.72 (95% CI 0.53–0.99); STFS: HR 2.45 (95% CI 1.48–4.08, *p* < 0.001)	Prognostic/Diagnostic/Potential predictive	Exploratory	[63]
VEGF	Serum	High levels associated with recurrence and poor survival	Serum VEGF > 416 pg/mL: reduced RFS (*p* = 0.003)	Prognostic/Potential predictive	Exploratory	[67]
IL-6	Serum	Associated with metastatic status, poor treatment outcome and shorter PFS/OS	PFS: HR 3.51 (*p* = 0.003); OS: HR 7.18 (*p* = 0.001); PFS: HR 1.68 (*p* = 0.0021); OS: HR 3.28 (*p* < 0.0001); PFS: HR 0.40 (*p* = 0.028)	Prognostic/Potential predictive	Exploratory	[80]
IL-8	Serum	High levels associated with reduced OS/PFS and poor patient outcome	NIVOREN: HR 2.57 (*p* < 0.001); PFS: HR 1.27 (95% CI 1.01–1.59, *p* = 0.037); OS: HR 1.85 (95% CI 1.21–2.84, *p* = 0.001)	Prognostic/Potential predictive	Exploratory	[68,81]
IL-10	Serum	High levels associated with poor responses to ICIs	Detectable in 13.2%	Prognostic/Potential predictive	Exploratory	[88,89]
sPD-L1/sPD-1	Serum/Plasma	Associated with metastatic disease and poor survival	sPD-L1 ≥ 42.6 pg/mL; lower 5-year OS (*p* = 0.0012); Plasma sPD-L1 > 793 ng/mL; poor survival	Prognostic/Potential predictive	[92]	[90]
sBTN3A1	Plasma	Associated with longer PFS and best OS	-	Potential predictive	[92]	[92]
sCD27	Plasma	sCD27 associated with poor OS and resistance to anti-PD-1 therapy	Plasma sCD27; OS: HR 5.02 (*p* = 0.004);	Prognostic/Potential predictive	[107]	[107]
sCD28/sCTLA-4	Plasma	Associated with poor clinical outcome	sCD28: rho = −0.33, *P* = 0.04	Potential prognostic	Exploratory	[95]
sCD80/sCD137	Plasma	Showed minimal circulation variation	-	Not established	Exploratory	[95]
sCD163	Serum	High levels associated with metastatic disease,	-	Prognostic/Potential diagnostic	Exploratory	[86]
sCD206	Serum	High levels associated with reduced OS	HR = 1.37, 95% CI: 1.12–1.67, *p* = 0.002	Potential prognostic	Exploratory	[108]
CA15.3/MUC1	Serum	Associated with nodal and visceral metastases, advanced tumor stage, and higher nuclear grade	Serum CA15.3: poorer cancer-specific survival (HR 1.97, 95% CI 1.08–2.94, *p* = 0.01)	Prognostic/Potential diagnostic	Exploratory	[111]
HSC71/HSPA8	Serum	Increased levels in ccRCC	AUC: 0.86 (95% CI: 0.76–0.96); sensitivity: 87% (95% CI: 69–96%); specificity: 80% (95% CI: 61–92%).	Diagnostic	Exploratory	[112]
SERPINA1/APOH	Urine	Elevated urinary levels reported in VHL-associated ccRCC	-	Potential diagnostic	Exploratory	[113]
C1QB/C1QC/ANXA1/LYZ/S100A9/SERPINA4	Serum	Associated with tumor stage and grade	-	Potential prognostic and diagnostic	Exploratory	[114]
sLAG-3	Plasma	Associated with increased risk of advanced disease	Advanced disease: OR 3.36 (95% CI 1.55–7.27, *p* = 0.002);	Potential prognostic	Exploratory	[95]
sTIM-3/sBTLA	Plasma	Associated with high risk of death	sTIM-3: OS HR 3.12 (95% CI 1.44–6.75, *p* = 0.0039); sBTLA: OS HR 6.02 (95% CI 2.00–18.10, *p* = 0.0014)	Prognostic	Exploratory	[95]
Hsp27	Serum/Urine	High levels associated with high-grade tumors	-	Potential diagnostic	Exploratory	[115]
HIG2	Plasma	Elevated levels in RCC	-	Potential diagnostic	Exploratory	[116]
CAIX	Plasma	High levels in ccRCC				[117]
IMP3	Plasma	High levels associated with distant metastases	-	Prognostic	Exploratory	[118]
HP/FN1/MGAT4A/APOA1/KNG1/UMOD/ APOD/CD59/HSPG2	Urine	Associated with poor prognosis				[119]
RALA/CNDP1	Urine	Associated with degree of venous invasion	-	Potential prognostic	Exploratory	[120]
LUM/CRNN/ANXA2	Urine	High levels associated with tumor grade	-	Potential prognostic	Exploratory	[120]
CD97/COCH/PIK3IP1	Urine	Associated with advancing tumor stage	-	Potential prognostic	Exploratory	[121]
APOB/FN1/ CERU/CFH/HP/PLTP	Urine	Decreased compared with healthy controls	-	Potential prognostic	Exploratory	[121]
GLRX/CST3/SLC9A3R1/HSPE1/FKBP1A/ EEF1G/C12orf49/EHD4/EPS8L2/CHMP2A/ PDCD6IP/CNDP2/CEACAM1/CCT6A	Urine	Probably detecting early-stage disease and predicting disease progression	-	Potential diagnostic/Prognostic	Exploratory	[122]

Abbreviations: ANXA1, annexin A1; ANXA2, annexin A2; APOA1, apolipoprotein A1; APOB, apolipoprotein B; APOD, apolipoprotein D; APOH, apolipoprotein H; BTLA, B- and T-lymphocyte attenuator; BTN3A1, butyrophilin subfamily 3 member A1; C1QB, complement C1q B chain; C1QC, complement C1q C chain; C12orf49, chromosome 12 open reading frame 49; CAIX, carbonic anhydrase IX; ccRCC, clear-cell renal cell carcinoma; CCT6A, chaperonin-containing TCP1 subunit 6A; CD, cluster of differentiation; CEACAM1, carcinoembryonic antigen-related cell adhesion molecule 1; CERU, ceruloplasmin; CFH, complement factor H; CHMP2A, charged multivesicular body protein 2A; CNDP1, carnosine dipeptidase 1; CNDP2, carnosine dipeptidase 2; COCH, cochlin; CRNN, cornulin; CST3, cystatin C; CTLA-4, cytotoxic T-lymphocyte-associated protein 4; DFS, disease-free survival; EEF1G, eukaryotic translation elongation factor 1 gamma; EHD4, EH-domain containing 4; EPS8L2, EPS8-like 2; FKBP1A, FKBP prolyl isomerase 1A; FN1, fibronectin 1; GLRX, glutaredoxin; HIG2, hypoxia-inducible protein 2; HP, haptoglobin; HSC71, heat shock cognate 71-kDa protein; Hsp27, heat shock protein 27; HSPA8, heat shock protein family A member 8; HSPE1, heat shock protein family E member 1; HSPG2, heparan sulfate proteoglycan 2; ICI, immune checkpoint inhibitor; IL, interleukin; IMP3, insulin-like growth factor II mRNA-binding protein 3; KIM-1, kidney injury molecule-1; KNG1, kininogen 1; LAG-3, lymphocyte-activation gene 3; LUM, lumican; LYZ, lysozyme; MGAT4A, mannosyl (α-1,3-)-glycoprotein β-1,4-N-acetylglucosaminyltransferase A; MUC1, mucin 1; OS, overall survival; PD-1, programmed cell death protein 1; PD-L1, programmed death-ligand 1; PDCD6IP, programmed cell death 6-interacting protein; PFS, progression-free survival; PIK3IP1, phosphoinositide-3-kinase-interacting protein 1; PLTP, phospholipid transfer protein; RALA, RAS-like proto-oncogene A; RCC, renal cell carcinoma; s, soluble; S100A9, S100 calcium-binding protein A9; SERPINA1, serpin family A member 1; SERPINA4, serpin family A member 4; SLC9A3R1, solute carrier family 9 member A3 regulator 1; STFS, systemic therapy-free survival; TIM-3, T-cell immunoglobulin and mucin-domain containing 3; UMOD, uromodulin; VEGF, vascular endothelial growth factor; and VHL, von Hippel–Lindau.

**Table 3:** Established and emerging immune checkpoint-targeted therapies in clear cell renal cell carcinoma (ccRCC).

Target	Drug	Mechanism of Antitumor Activity	FDA Approval	Representative Clinical Trial and ID	Current Status	References
PD-1	Nivolumab	Restores T-cell activity and enhances antitumor immunity	Established: nivolumab + ipilimumab; nivolumab + cabozantinib; prior-line nivolumab	CheckMate 025 (NCT01668784)	Standard therapy	[126]
PD-1	Pembrolizumab	Restores T-cell activity and enhances antitumor immunity	Established: pembrolizumab + axitinib; pembrolizumab + lenvatinib; adjuvant pembrolizumab; adjuvant pembrolizumab + belzutifan	KEYNOTE-427 (NCT02853344)	Standard therapy	[44]
PD-L1	Atezolizumab	Restores T-cell-mediated antitumor immunity	Not an FDA-approved RCC regimen	IMmotion150 (NCT01984242)	Investigational	[44]
PD-L1	Avelumab	Restores T-cell-mediated antitumor immunity	Established: avelumab + axitinib	JAVELIN Renal 101 (NCT02684006)	Standard therapy	[24]
CTLA-4	Ipilimumab	Enhances T-cell priming and activation	Established in combination with nivolumab	CheckMate 214 (NCT02231749)	Standard therapy	[129]
LAG-3	Relatlimab	Reverses T-cell exhaustion and enhances antitumor immunity	Not an FDA-approved RCC regimen	NCT06708949	Investigational	ClinicalTrials.gov
TIGIT	Tiragolumab	Enhances T-cell and NK cell-mediated antitumor immunity	Not an FDA-approved RCC regimen	NCT05805501	Investigational	ClinicalTrials.gov
IDO-1	Epacadostat	Reduces tumor-induced immunosuppression	Not an FDA-approved RCC regimen	KEYNOTE-679 (NCT03260894)	Investigational/negative phase III development	[133]
ENPP3xCD3	XmAb819	Redirects and activates T-cells against ENPP3-expressing tumor cells	Not an FDA-approved RCC regimen	NCT05433142	Investigational	[135]
CD70	ALLO-316	Targets/Eliminates CD70-expressing tumor cells	Not an FDA-approved RCC regimen	TRAVERSE (NCT04696731)	Investigational	[137]
HERV-E	HERV-E TCR-engineered T cells	Promotes tumor regression	Not an FDA-approved RCC regimen	NCT03354390	Investigational	[138]

Abbreviations: CD3, cluster of differentiation 3; CD70, cluster of differentiation 70; CTLA-4, cytotoxic T-lymphocyte-associated protein 4; ENPP3, ectonucleotide pyrophosphatase/phosphodiesterase 3; HERV-3, human endogenous retrovirus type E; IDO-1, indoleamine 2,3-dioxygenase 1; LAG-3, lymphocyte-activation gene 3; NK cell, natural killer cell; PD-1, programmed cell death protein 1; PD-L1, programmed death-ligand 1; TCR, T-cell receptor; and TIGIT, T-cell immunoreceptor with immunoglobulin and ITIM domains.

**Table 4:** Combination therapy trials involving dual immune checkpoint inhibitors (ICIs) or ICIs with vascular endothelial growth factor (VEGF) therapies in advanced clear cell renal cell carcinoma (ccRCC) or advanced renal cell carcinoma (RCC) with a clear-cell component.

Combination	Therapeutic Class	Clinical Trial and ID	Phase	Comparator	FDA Approval/Year	Administration Route	References
Nivolumab + Ipilimumab	Dual ICI	CheckMate 214 (NCT02231749)	III	Sunitinib	Yes/2018	Intravenous	[37]
Nivolumab + cabozantinib	ICI + VEGF-TKI	CheckMate 9ER (NCT03141177)	III	Sunitinib	Yes/2021	Intravenous + oral	[141]
Pembrolizumab + Axitinib	ICI + VEGF-TKI	KEYNOTE-426 (NCT02853331)	III	Sunitinib	Yes/2019	Intravenous + oral	[142]
Avelumab + Axitinib	ICI + VEGF-TKI	JAVELIN Renal 101 (NCT02684006)	III	Sunitinib	Yes/2019	Intravenous + oral	[144]
Pembrolizumab + Lenvatinib	ICI + VEGF-TKI	CLEAR/KEYNOTE -581 (NCT02811861)	III	Sunitinib	Yes/2021	Intravenous + oral	[44]
Atezolizumab + Bevacizumab	ICI + VEGF antibody	IMmotion 151 (NCT02420821)	III	Sunitinib	No	Intravenous	[146]

Abbreviations: ICI, immune checkpoint inhibitors; VEGF-TKI, vascular endothelial growth factor tyrosine kinase inhibitors.

**Table 5:** Emerging combination strategies for advanced renal cell carcinoma (RCC) with clear-cell histology.

Combination	Therapeutic Class	Clinical Trial and ID	Phase	Comparator	Administration Route	References
Relatlimab + nivolumab + ipilimumab	Triple immune checkpoint blockade	NCT06708949	II	Nivolumab + ipilimumab	Intravenous	[140]
Nemvaleukin alfa + pembrolizumab	ICI + cytokine	ARTISTRY-1 (NCT02799095)	I/II	-	Intravenous	[148]
Pegilodecakin + Pembrolizumab or nivolumab	ICI + cytokine	IVY (NCT02009449)	I/Ib	-	Subcutaneous + Intravenous	[149]
Bempegaldesleukin + nivolumab	ICI + cytokine	PIVOT-02 (NCT02983045)/PIVOT-09 (NCT03729245)	I (PIVOT-02)/III (PIVOT-09)	TKI-based therapy	Intravenous	[151,152]
Toripalimab + axitinib	ICI + VEGF-TKI	RENOTORCH (NCT04394975)	III	Sunitinib	Intravenous	ClinicalTrials.gov
Sitravatinib + nivolumab	ICI + TKI	SNAPI (NCT04904302)	II	Sitravatinib	Oral + intravenous	ClinicalTrials.gov
Cabozantinib + nivolumab + ipilimumab	Triple therapy + VEGF -TKI)	COSMIC-313 (NCT03937219)	III	Nivolumab + ipilimumab	Oral + intravenous + intravenous	ClinicalTrials.gov
Telaglenastat + everolimus	Metabolic inhibitor + mTOR inhibitor	ENTRATA (NCT03163667)	II	Placebo + everolimus	Oral	[153]
Telaglenastat + cabozantinib	Metabolic inhibitor + VEGF-TKI	CANTATA (NCT03428217)	II	Placebo + cabozantinib	Oral	[153]

Abbreviations: ICI, immune checkpoint inhibitors; VEGF-TKI, vascular endothelial growth factor tyrosine kinase inhibitors.

## Data Availability

No new data were created or analyzed in this study. Data sharing is not applicable to this article.

## Supplementary Materials

The following supporting information can be downloaded at: https://www.mdpi.com/article/10.3390/ijms27219764/s1.

## Abbreviations

The following abbreviations are used in this manuscript:

^68^Ga-PSMA PET/CT	gallium-68 prostate-specific membrane antigen positron emission tomography/computed tomography
AKT	protein kinase B
ANXA1	annexin A1
ANXA2	annexin A2
APOA1	apolipoprotein A1
APOB	apolipoprotein B
APOD	apolipoprotein D
APOH	apolipoprotein H
C1QB	complement C1q B chain
C1QC	complement C1q C chain
C12orf49	chromosome 12 open reading frame 49
CA15.3	cancer antigen 15.3
CAIX	carbonic anhydrase IX
CAR	chimeric antigen receptor
ccRCC	clear cell renal cell carcinoma
CCT6A	chaperonin containing TCP1 subunit 6A
CD3	cluster of differentiation 3
CD4+	cluster of differentiation 4+
CD8+	cluster of differentiation 8+
CD27	cluster of differentiation 27
CD28	cluster of differentiation 28
CD59	cluster of differentiation 59
CD70	cluster of differentiation 70
CD80	cluster of differentiation 80
CD97	cluster of differentiation 97
CD137	cluster of differentiation 137
CD155	cluster of differentiation 155
CD163	cluster of differentiation 163
CD206	cluster of differentiation 206
CEACAM1	CEA cell adhesion molecule 1
CERU	ceruloplasmin
CETP	cholesteryl ester transfer protein
CFH	complement factor H
CHMP2A	charged multivesicular body protein 2A
chRCC	chromophobe renal cell carcinoma
CI	confidence interval
CNDP1	carnosine dipeptidase 1
CNDP2	carnosine dipeptidase 2
COCH	cochlin
COX5B	cytochrome c oxidase subunit 5B
CRNN	cornulin
CSS	cancer-specific survival
CST3	cystatin C
ctDNA	circulating tumor DNA
CTLA-4	cytotoxic T-lymphocyte-associated protein 4
CXCL8	C-X-C motif chemokine ligand 8
CXCL10	C-X-C motif chemokine ligand 10
DFS	disease-free survival
DMR	distant metastatic relapse
DNASE1L3	deoxyribonuclease 1-like 3
DSS	disease-specific survival
EEF1G	eukaryotic translation elongation factor 1 gamma
EHD4	EH domain containing 4
EMT	epithelial–mesenchymal transition
ENPP3	ectonucleotide pyrophosphatase/phosphodiesterase 3
EPS8L2	EPS8 like 2
FDA	U.S. Food and Drug Administration
FGFBP2	fibroblast growth factor binding protein 2
FGFR1	fibroblast growth factor receptor 1
FKBP1A	FKBP prolyl isomerase 1A
FN1	fibronectin 1
GLRX	glutaredoxin
GOT1	glutamic-oxaloacetic transaminase 1
HERV-E	human endogenous retrovirus type E
HIF-1α	hypoxia-inducible factor 1 alpha
HIF-2α	hypoxia-inducible factor 2 alpha
HIG2	hypoxia-inducible protein 2
HP	haptoglobin
HR	hazard ratio
HSC71/HSPA8	heat shock cognate 71 kDa protein/heat shock protein family A (Hsp70) member 8
Hsp27	heat shock protein 27
HSPE1	heat shock protein family E (Hsp10) member 1
HSPG2	heparan sulfate proteoglycan 2
ICI	immune checkpoint inhibitor
IDO-1	indoleamine 2,3-dioxygenase 1
IFN-α	interferon-α
IFN-γ	interferon-γ
IgG1	immunoglobulin G1
IgG4	immunoglobulin G4
IHC	immunohistochemistry
IL-2	interleukin-2
IL-6	interleukin-6
IL-8	interleukin-8
IL-10	interleukin-10
IL-27	interleukin-27
IMDC	International Metastatic Renal Cell Carcinoma Database Consortium
IMP3	insulin-like growth factor II mRNA-binding protein 3
iTRAQ	isobaric tags for relative and absolute quantitation
KIM-1	kidney injury molecule-1
KNG1	kininogen 1
LAG-3	lymphocyte-activation gene 3
LUM	lumican
LYZ	lysozyme
MASS	morphology, attenuation, size, and structure
mccRCC	metastatic clear cell renal cell carcinoma
MGAT4A	alpha-1,3-mannosyl-glycoprotein 4-beta-N-acetylglucosaminyltransferase A
MRD	minimal residual disease
mTOR	mechanistic target of rapamycin
MUC1	mucin 1
MZT2A	mitotic spindle organizing protein 2A
NK	natural killer
NSCLC	non-small-cell lung cancer
OR	odds ratio
ORR	objective response rate
OS	overall survival
PD-1	programmed cell death protein 1
PD-L1	programmed death-ligand 1
PD-L2	programmed death-ligand 2
PDCD6IP	programmed cell death 6 interacting protein
PFS	progression-free survival
PI3K	phosphoinositide 3-kinase
PIK3IP1	phosphoinositide-3-kinase interacting protein 1
PLTP	phospholipid transfer protein
pRCC	papillary renal cell carcinoma
RALA	RAS like proto-oncogene A
RCC	renal cell carcinoma
RFS	recurrence-free survival
RTK	receptor tyrosine kinase
S100A9	S100 calcium binding protein A9
sBTLA	soluble B- and T-lymphocyte attenuator
sBTN3A1	soluble butyrophilin subfamily 3 member A1
sCD27	soluble CD27
sCD28	soluble CD28
sCD163	soluble CD163
sCD206	soluble CD206
sCTLA-4	soluble CTLA-4
SERPINA1	serpin family A member 1
SERPINA4	serpin family A member 4
SEZ6L2	seizure related 6 homolog like 2
sLAG-3	soluble LAG-3
SLC9A3R1	solute carrier family 9 member A3 regulator 1
sPD-1	soluble PD-1
sPD-L1	soluble PD-L1
sPD-L2	soluble PD-L2
STAT3	signal transducer and activator of transcription 3
STFS	systemic therapy–free survival
sTIM-3	soluble TIM-3
TCGA	The Cancer Genome Atlas
TIGIT	T-cell immunoreceptor with immunoglobulin and ITIM domains
TILs	tumor-infiltrating lymphocytes
TIM-3	T-cell immunoglobulin and mucin-domain containing-3
TIMCs	tumor-infiltrating mononuclear cells
TKI	tyrosine kinase inhibitor
TME	tumor microenvironment
TNM	tumor-node-metastasis
UMOD	uromodulin
VEGF	vascular endothelial growth factor
VEGFR2	vascular endothelial growth factor receptor 2
VHL	von Hippel–Lindau
VISTA	V-domain immunoglobulin suppressor of T-cell activation
WHO	World Health Organization
