# Supplementary material for: Immune-Related Proteomic Profiling in Clear Cell Renal Cell Carcinoma: Tissue and Soluble Biofluid Biomarkers
Source: Int J Mol Sci. 2026 Oct 30;27(21):9764. doi: 10.3390/ijms27219764 (PMC13762477; doi:10.3390/ijms27219764)
Supplement: Supplementary file 1 [file ijms-27-09764-s001.zip › ijms-4640196-supplementary.pdf]

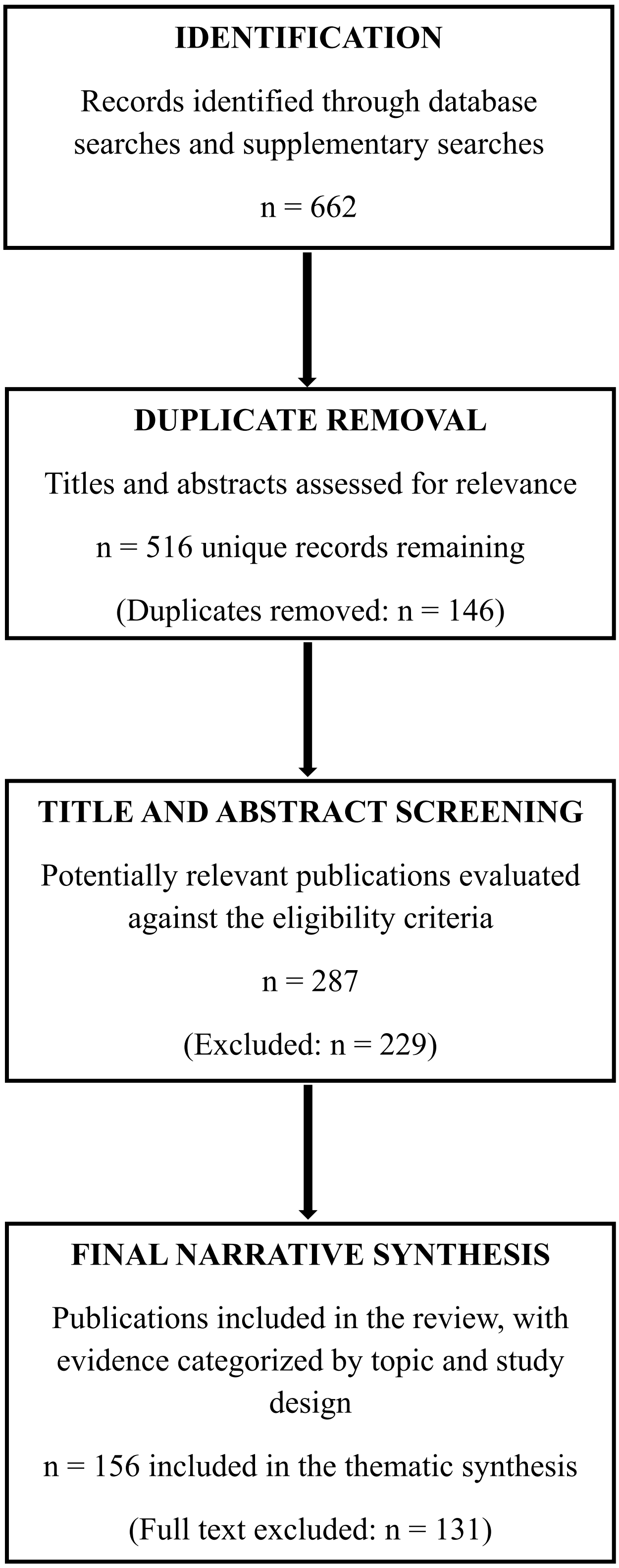

Supplementary Figure S1. Flow diagram of the literature search and selection process. The diagram illustrates the identification, screening, full-text assessment, and final selection of publications included in the narrative review of immune-related proteomic biomarkers in ccRCC. The selected literature was categorized according to topic and study design to support the narrative synthesis. This figure documents the audit trail of a structured narrative review and should not be interpreted as a PRISMA flow diagram or a fully reproducible systematic-review search process.
